# An adverse outcome pathway for cigarette smoke-mediated oxidative stress in plaque formation

**DOI:** 10.3389/ftox.2025.1554747

**Published:** 2025-06-30

**Authors:** Patrudu Makena, Linsey E. Haswell, Michael McEwan, Brian M. Keyser, David E. Smart, Robert Leverette, Kristen Jordan, Damien Breheny, Sarah Baxter-Wright

**Affiliations:** ^1^ RAI Services Company, Winston-Salem, NC, United States; ^2^ Research and Development, British American Tobacco (Investments) Limited, Southampton, United Kingdom

**Keywords:** cigarette smoking, atherosclerosis, CVD, oxidative stress, inflammation, reactive oxygen species, AOP

## Abstract

Adverse outcome pathways (AOPs) have been developed as a risk assessment tool for regulatory applications. These AOPs describe a logical mechanistic sequence of events, starting with a Molecular Initiating Event (MIE), and ultimately leading to a disease outcome via a series of Key Events (KE). The AOP framework provides a system to make predictions and assessments while reducing the need for *in vivo* assessment. In the absence of epidemiological evidence, assessment of the health effects of a product, chemical or therapy on the progression of atherosclerosis would necessitate long-term animal exposure studies such as the use of the Apolipoprotein E deficient mouse. We followed Organisation for Economic Co-operation and Development (OECD) guidelines to formulate and propose an AOP for atherosclerotic plaque progression, collating the evidence by which cigarette smoke-induced oxidative stress forms a MIE. The downstream pathway includes multiple KEs including the upregulation of proinflammatory mediators, nitric oxide depletion, and endothelial dysfunction. Alterations in these KEs can lead to plaque formation and progression in cardiovascular disease and increase the risk of morbidity and mortality. Identifying preclinical endpoints and clinical biomarkers associated with these KEs provides a framework for *in vitro* and clinical data, supporting a mechanistic narrative for regulatory assessment. The application of this pathway provides a powerful alternative to animal models through developing preclinical assays and biomarkers for the assessment of atherosclerosis progression risk.

## Introduction

Atherosclerosis is a thickening and loss of arterial wall elasticity that occurs with the formation of atherosclerotic plaques within the arterial intima (Noyes and Thompson, 2014). Atherosclerosis is a chronic arterial disease; atherosclerotic plaque rupture and thrombosis are the main cause of the majority of acute coronary syndromes and sudden coronary death (Sakakura et al., 2013; Herrington et al., 2016). The main clinical manifestations of atherosclerosis include ischemic heart disease, ischemic stroke, and peripheral arterial disease (Herrington et al., 2016). Ischemic heart disease (most commonly due to atherosclerosis of the coronary arteries) and stroke are the two leading causes of death in the world (Libby et al., 2019).

The etiology of atherosclerosis is generally unknown, but there are multiple factors that contribute to atherosclerotic plaque progression. These include genetic and acquired factors. Today atherosclerosis is considered an inflammatory process that occurs as a response to the accumulating lipid within the arterial wall (Feingold et al., 2000). Increase in plasma cholesterol levels result in accumulation of lipids, especially “cholesterol-containing low-density lipoproteins (LDL)” into the arterial wall where they bind to the extracellular matrix and aggregate (Sakakura et al., 2013; Arai, 2014; Feingold et al., 2000; Orekhov, 2018). Also, circulating monocytes adhere to the endothelial cells and populate the subendothelial space (Sakakura et al., 2013). The monocytes then differentiate to macrophages and subsequently convert to foamy macrophages (Scott, 1977; Yu et al., 2013; Chistiakov et al., 2017; Orekhov, 2018).

Foamy macrophage infiltration of pathological intimal thickening (PIT) is considered the first step towards the eventual formation of atherosclerotic plaque (Sakakura et al., 2013). Mechanisms involved in atherosclerotic plaque progression from PIT to fibroatheromas include further infiltration of macrophages and foam cells, vascular inflammation, oxidative stress and subsequent endothelial dysfunction (Schächinger and Zeiher, 2000; Alp et al., 2004; Sakakura et al., 2013; Otsuka et al., 2015; Gimbrone and García-Cardeña, 2016; Otsuka et al., 2016; Förstermann et al., 2017; Chen et al., 2018). Further infiltration of macrophages, which releases matrix metalloproteinase (MMPs), along with macrophage and vascular smooth muscle cell apoptosis accompanied with the intraplaque hemorrhage, leads to the formation and expansion of an acellular necrotic core (Sakakura et al., 2013). The precursor lesion of plaque rupture is a thin cap fibroatheroma (TCFA) or “vulnerable plaque” (Sakakura et al., 2013; Otsuka et al., 2016). Atherosclerotic plaque rupture and erosion leads to the activation of blood coagulation cascade and results in luminal thrombosis. Atherosclerotic plaque disruption with superimposed thrombosis is called atherothrombosis, a term that includes both atherosclerosis and its acute thrombotic complications (Viles-Gonzalez et al., 2004). Atherosclerotic and thrombotic processes are interdependent but thrombosis is not an obligatory consequence of atherosclerosis (Viles-Gonzalez et al., 2004).

There are many known risk factors for atherosclerosis, including hypercholesterolemia, hypertension, diabetes, and smoking, which are involved in the pathogenesis of atherosclerosis (Fan and Watanabe, 2022). Of the various risk factors, cigarette smoking is a major preventable risk factor for cardiovascular disease that directly affects atherosclerosis (Messner and Bernhard, 2014; Siasos et al., 2014; Wang et al., 2021).

Cigarette smoke is a complex aerosol mixture that contains thousands of chemicals including reactive aldehydes, polycyclic hydrocarbons, and quinones, as well as reactive oxygen and nitrogen species (ROS/RNS) that can trigger cellular oxidative stress either directly or through activation of cellular oxidative stress signaling pathways (Takajo et al., 2001; Ambrose and Barua, 2004; Orosz et al., 2007; Yamaguchi et al., 2007; Abdelghany et al., 2018; Wang et al., 2019; El-Mahdy et al., 2020).

Oxidative stress alters the redox state of an organism and creates an imbalance between production of ROS and endogenous antioxidant defenses, leading to oxidation of lipids, proteins, and DNA (Checa and Aran, 2020). ROS are produced from molecular oxygen as a result of normal cellular metabolism. ROS can be divided into two groups: free radicals and nonradicals. The main ROS that are of physiological significance include O_2_^−^ and H_2_O_2_ (nonradical), and ONOO^−^ (Burke and Fitzgerald, 2003; Birben et al., 2012). ROS are mainly produced by mitochondria, during both pathological and physiological conditions such as cellular respiration, during arachidonic acid metabolism, and by endothelial and inflammatory cells (Pizzino et al., 2017). At the low physiological level, ROS regulate many essential processes like protein phosphorylation, activation of several transcriptional factors, apoptosis, immunity, and differentiation (Rajendran et al., 2014; Zhang et al., 2016). A large body of evidence shows that oxidative stress responsible in the onset and progression of several diseases such as cancer, diabetes, metabolic disorders, atherosclerosis, atherothrombosis, and cardiovascular diseases (Pizzino et al., 2017; Kattoor et al., 2017; Martin-Ventura et al., 2017; Checa and Aran, 2020).

In this manuscript, we present an AOP for cigarette smoke-induced atherosclerosis through oxidative stress mediated atherosclerotic plaque formation.

### AOP development process

AOP is a simplified linear framework that originates from the interaction of any external stressor with a biological target that initiates a molecular initiating event (MIE) and launches the sequence of consecutive key events (KEs) at different levels of biological organization connected by key event relationships (KERs), and subsequently leading to an adverse outcome (AO) (Users’ Handbook supplement to the guidance document for developing and assessing Adverse Outcome Pathways (OECD, 2013). MIE is a specialized type of key event that represents the initial point of chemical/stressor interaction at the molecular level within the organism, resulting in a perturbation that starts the AOP (Villeneuve et al., 2014a; Villeneuve et al., 2014b; Vinken et al., 2017). A KE is a change in biological or physiological state that is both measurable and essential to the progression of a defined biological perturbation leading to a specific AO (Villeneuve et al., 2014a; Villeneuve et al., 2014b; Vinken et al., 2017). KER is a relationship that connects 1 KE to another, and defines a causal and predictive relationship between the upstream and downstream event (Villeneuve et al., 2014a; Villeneuve et al., 2014b; Vinken et al., 2017). AO is a specialized type of key event that is generally accepted as being of regulatory significance on the basis of correspondence to an established protection goal or equivalence to an apical endpoint in an accepted regulatory guideline toxicity test (Villeneuve et al., 2014a; Villeneuve et al., 2014b; Vinken et al., 2017).

An AOP describes existing knowledge about the exposure, molecular, cellular, tissue, organ, organism, and population perturbations initiated by a stressor that leads to eventual toxicological effect (AO) at a biological level of organization relevant to risk assessment. The (OECD, 2013) guidelines were employed for the development of this AOP (Users’ Handbook supplement to the Guidance Document for developing and assessing Adverse Outcome Pathways). The guidance documents and subsequent supplements to the guidance identify key information to include in an AOP description and the AOP-KB (www.aopwiki.org) provides a structured, collaborative platform for assembling and disseminating AOP descriptions.

An AOP development process starts by identifying the stressor or chemical compounds or compound classes that have been experimentally proven to induce the specific AO. In-depth analysis of reliable literature was applied to establish a MIE, AO, and a series of specific KEs, which represent the essential intermediary steps in between the MIE and AO (Villeneuve et al., 2014a; Villeneuve et al., 2014b).

The assessment of the weight of evidence supporting the AOP was evaluated according to three principles, namely, essentiality of the KEs, biological plausibility of the KERs, and KERs empirical support (quantitative evidence) (Villeneuve et al., 2014b).

KE included in AOP must be essential, i.e., KE should be included in the single AOP only if measured responses are involved in the AOP progression toward the certain single AO, and causally associated with MIE or upstream/downstream KEs. KE is considered essential if its blocking prevents all downstream KEs and/or AO. KER biological plausibility defines the mechanistic (i.e., structural or functional) relationship between upstream and downstream KE consistent with established biological knowledge. KER biological plausibility is evaluated with respect to current understanding of normal biology, rather than response to specific stressor. KER empirical support is evaluated with regards to specific experimental evidence that supports the associations between pairs of upstream and downstream KEs, i.e., it must be experimentally shown that a change in an upstream KE leads to the appropriate change in the downstream KE. It is examined most often in studies of dose-response/incidence and temporal relationships for stressors that initiate the AOP, thereby the articles selected for KER empirical support overview must be stressor- and AO-specific (Villeneuve et al., 2014a; Villeneuve et al., 2014b).

### AOP MIE, KEs and AO

**FIGURE 1:**
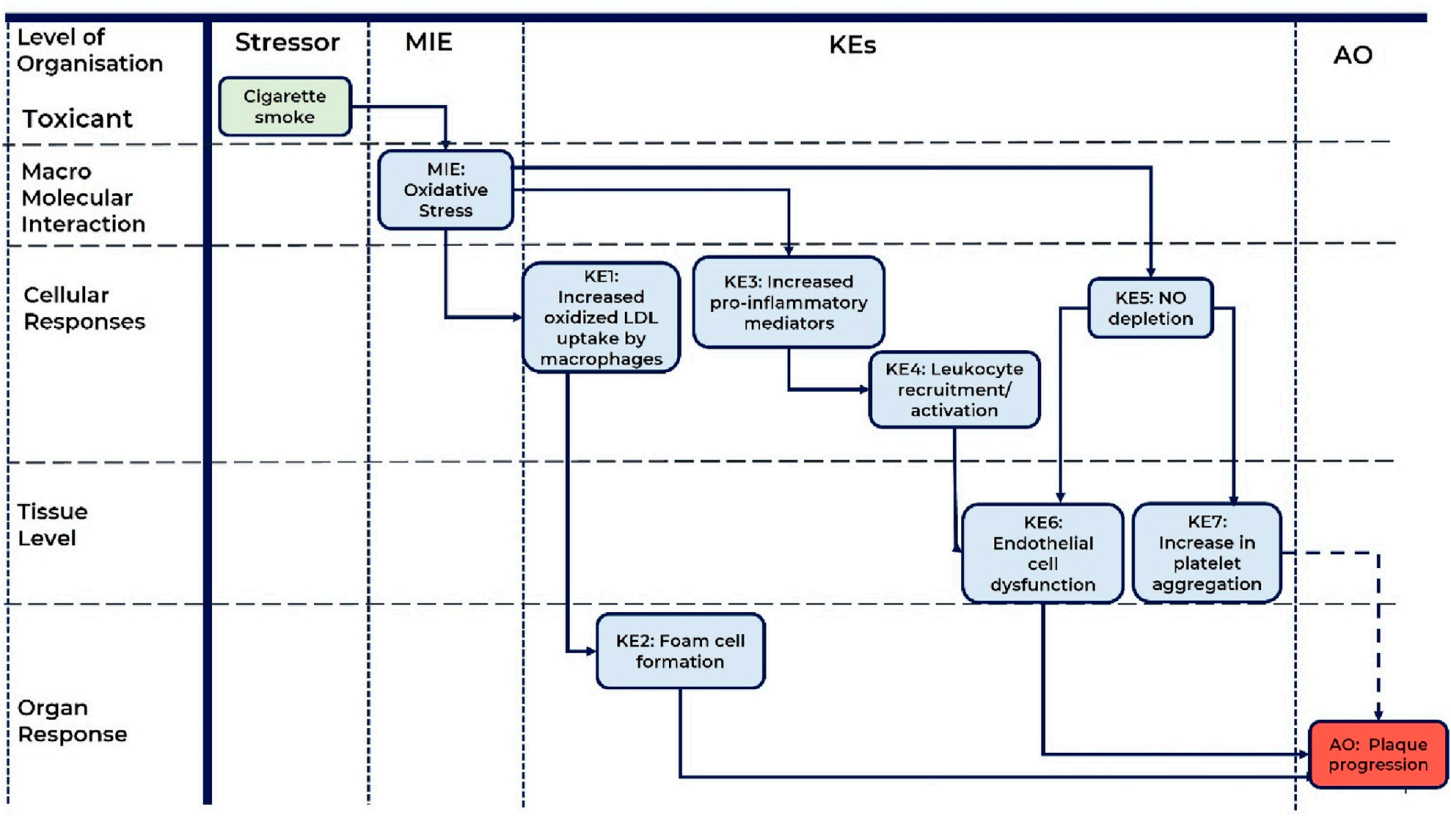
A schematic representation of the AOP: Role of Oxidative Stress induced by cigarette smoke in atherosclerotic plaque progression. MIE, molecular initiating event; KE, key event; AO, adverse outcome; KER, key event relationship; LDL, low density lipoproteins; NO, nitric oxide. Solid line arrow corresponds to direct KER, dashed line arrow corresponds to indirect KER.

A schematic representation of the AOP is presented in Figure 1.

### MIE: oxidative stress

At the molecular level of biological organization, oxidative stress was proposed as the MIE of this AOP. There is adequate evidence that inhalation of toxicants/oxidants including cigarette smoke significantly increases the risk for atherosclerosis progression (Howard et al., 1998; Siasos et al., 2014; Babic et al., 2019; Centner et al., 2020; Wang et al., 2021). The key mechanism through which inhaled toxicants/oxidants aggravate atherosclerosis is induction of oxidative stress in the vascular endothelium (Ambrose and Barua, 2004; Messner and Bernhard, 2014; Siasos et al., 2014; Förstermann et al., 2017; El-Mahdy et al., 2020; Wang et al., 2021). Oxidative stress arises from cigarette smoke-derived oxidants (gas and tar phase of cigarette smoke) and cigarette smoke-induced production of reactive oxygen species (ROS) by vascular resident cells (endothelial cells, smooth muscle cells) and infiltrating cells (platelets, monocytes/macrophages) (Takajo et al., 2001; Ambrose and Barua, 2004; Orosz et al., 2007; Yamaguchi et al., 2007; Zhou et al., 2013; Wang et al., 2019; El-Mahdy et al., 2020). Therefore, oxidative stress plays a central role in smoking-induced atherosclerosis progression, hence it was considered as MIE for this AOP (Harrison et al., 2003; Förstermann et al., 2017; Malekmohammad et al., 2019).

### KE1: increased oxidized LDL uptake by macrophages

At the level of cellular response, cigarette smoke-induced oxidative stress significantly increases lipid peroxidation resulting in an increase in atherogenic oxidized-low density lipoproteins (ox-LDL) levels (Scheffler et al., 1992; Frei et al., 1991; Morrow et al., 1995; Churg and Cherukupalli, 1993; Yamaguchi et al., 2005; Kunitomo et al., 2009; Messner and Bernhard, 2014). Moreover, oxidative stress increases expression of macrophage scavenger receptors which mediate recognition and uptake of ox-LDL leading to ox-LDL accumulation in macrophages and subsequent foam cell formation (Zhou et al., 2013; Feingold et al., 2000; Orekhov, 2018).

### KE2: foam cell formation

At the level of organ response, cigarette smoke induces differentiation of monocytes into macrophages. Macrophages accumulate lipids and differentiate into foam cells to form the early lesions that mature into atherosclerotic plaques (Zhou et al., 2013; Mehta and Dhawan, 2020). Activated macrophages and lipid-laden foam cells are considered to be the hallmarks of atherosclerotic plaques (Orekhov, 2018). Differentiation of monocytes, macrophages and foam cells are the key stages in atherosclerotic plaque development (Yu et al., 2013; Chistiakov et al., 2017; Orekhov, 2018).

### KE3: increased pro-inflammatory mediators

This KE is at the level of cellular response within AOP. Cigarette smoke-mediated oxidative stress promotes increased secretion and release of proinflammatory mediators in the vascular endothelium (Ambrose and Barua, 2004; Orosz et al., 2007; Messner and Bernhard, 2014). Oxidative stress launches several mechanisms in endothelial cells, macrophages, and vascular smooth muscle cells. In endothelial cells and macrophages, oxidative stress activates intracellular signaling pathways leading to increased transcription of several inflammatory factors, such as iNOS, TNF-alpha, IL-6, and IL-1 β (Brand et al., 1997; Orosz et al., 2007; Morgan and Liu, 2011; Checa and Aran, 2020; Zhang et al., 2016; Youn et al., 2016). Also, oxidative stress activates NALP3 inflammasome mechanism in endothelial cells (Schroder and Tschopp, 2010; Latz, 2010; Wu et al., 2018; Qian et al., 2021), monocytes/macrophages (Zhou et al., 2010; Mehta and Dhawan, 2020; Mehta et al., 2020a; Mehta et al., 2020b; Mao et al., 2021), and vascular smooth muscle cells (Grebe et al., 2018; Yao et al., 2019). NALP3 inflammasome activation leads to Caspase-1-mediated cell pyroptosis, a cell death mechanism that results in cell lysis (Qian et al., 2021). Also, activated Caspase-1 cleaves IL-18 and IL-1 β, thereby processing mature inflammatory cytokines IL-18 and IL-1 β. Endothelial cell, monocytes/macrophage, and vascular smooth muscle cell lysis leads to increased cytokine release (Miao et al., 2011; Wu et al., 2018; Qian et al., 2021; Grebe et al., 2018; Yao et al., 2019).

### KE4: leukocyte recruitment/activation

This KE belongs to the cellular response level of biological organization. The vascular inflammatory response is a characteristic hallmark of the initiation and progression of atherosclerosis (Ambrose and Barua, 2004; Tousoulis et al., 2016). Cigarette smoke-induced oxidative stress causes increased secretion of proinflammatory mediators and promotes vascular inflammation followed by monocyte/macrophage migration and recruitment to early atherosclerotic plaques (Orosz et al., 2007; Edirisinghe et al., 2008; Mao et al., 2021).

### KE5: NO depletion

This KE is at the cellular response level of biological organization. Nitric oxide (NO) is a soluble gas synthesized from the amino acid L-arginine in endothelial cells by the enzyme endothelial nitric oxide synthase (eNOS). NO plays an important role in the normal endothelial function and vascular homeostasis, including modulation of vascular tone, regulation of local cell growth, inhibition of platelet aggregation and protection of the vessel from injurious consequences of platelets and cells circulating in blood (Tousoulis et al., 2012). Moreover, NO exerts multiple anti-atherosclerotic effects (Sukhovershin et al., 2015; Förstermann et al., 2017).

One of the most important and well-studied consequences of oxidative stress caused by cigarette smoking is NO depletion in endothelial cells and platelets (Ichiki et al., 1996; Barua et al., 2003; Puranik and Celermajer, 2003; Grassi et al., 2010; Abdelghany et al., 2018; El-Mahdy et al., 2020). Augmented oxidative stress affects eNOS, promoting so-called eNOS ‘uncoupling’ (Heitzer et al., 2000; Abdelghany et al., 2018; El-Mahdy et al., 2020), which disturbs normal eNOS activity and suppresses NO synthesis via several different molecular mechanisms which are summarized in the “Pathways and molecular mechanisms“ section below (Takajo et al., 2001; Barua et al., 2003; Jin et al., 2003; Jaimes et al., 2004; Edirisinghe et al., 2008; Talib et al., 2014; Abdelghany et al., 2018; El-Mahdy et al., 2020). Oxidative stress-induced NO depletion subsequently leads to the development of vascular endothelial dysfunction and increased platelet aggregation; thereby playing a significant role in the progression of atherosclerosis (Alp et al., 2004; Li et al., 2018; Wang et al., 2021; El-Mahdy et al., 2020; Dikalov et al., 2019; Takajo et al., 2001).

### KE6: endothelial cell dysfunction

KE6 Endothelial dysfunction represents the tissue level of biological organization in this AOP. In the context of smoking, endothelial dysfunction can arise as a result of oxidative stress-induced NO depletion (Puranik and Celermajer, 2003; Alp et al., 2004; Chen et al., 2018; Li et al., 2018; Wang et al., 2021; El-Mahdy et al., 2020; Dikalov et al., 2019; Takajo et al., 2001). Endothelial dysfunction is characterized by impairment of endothelium-dependent relaxation and endothelial cell injury leading to atherosclerotic plaque formation (Siasos et al., 2014; Sukhovershin et al., 2015; Messner and Bernhard, 2014; Chen et al., 2018; Münzel et al., 2020; Wang et al., 2021). KE6 Endothelial dysfunction represents the tissue level of biological organization in this AOP.

### KE7: increased platelet aggregation

This KE lays at the tissue level of biological organization in this AOP. Platelet aggregation is the process by which platelets adhere to each other at sites of vascular injury leading to hemostatic plug formation and subsequent thrombosis (Jackson, 2007). Platelet aggregation plays a key role in pathogenesis of atherothrombosis, an acute complication of atherosclerosis (Ruggeri, 2002). Platelets adhere to the sites of vascular endothelial injury upon plaque rupture, become activated and aggregate to form a hemostatic thrombus (Ruggeri, 2002; Viles-Gonzalez et al., 2004; Steinhubl and Moliterno, 2005; Csordas and Bernhard, 2013; Martin-Ventura et al., 2017). Also, there are some data that demonstrate that platelet activation can be seen in the different phases of atherosclerosis, activated platelets are able to interact with endothelium and influence the development and progression of atherosclerotic plaque (Huo and Ley, 2004; Gawaz et al., 2008; Wang and Tang, 2020). Smoking-induced oxidative stress causes NO depletion leading to augmentation of platelet aggregation thereby likely contributing to the atherosclerotic plaque formation and atherothrombosis (Ichiki et al., 1996; Takajo et al., 2001).

### AO: plaque progression

Plaque progression was proposed as the AO for this AOP and represents the organ response level. Atherosclerotic plaque progression is a dynamic process involving the succession of early lesions to advanced plaques. The earliest stage of atherosclerotic lesions is termed pathologic intimal thickening (PIT), where the progressive lesion is primarily composed of layers of smooth muscle cells in a proteoglycan-collagen matrix characterized by extracellular lipid accumulation that are rich in proteoglycans and hyaluronan. Inflammation plays a necessary role in the progression of atherosclerotic lesions. The extensive vascular inflammation and infiltration of macrophages and foam cells result in progression of PIT to fibroatheromas; however, the processes involved are poorly understood (Sakakura et al., 2013; Otsuka et al., 2016). Fibroatheroma is characterized by the presence of an acellular necrotic core which is made up of cellular debris (Otsuka et al., 2015). The further expansion of the necrotic core along with the thinning of the fibrous cap leads to development of thin-cap fibroatheroma (TCFA) or ‘vulnerable’ plaque which is the precursor lesion of plaque rupture (Sakakura et al., 2013; Otsuka et al., 2016). Plaque ruptures is when the plaque fibrous cap becomes weakened and finally disrupted (Otsuka et al., 2016). When the fibrous cap ruptures and the necrotic core contents are released to the blood, the coagulation cascade becomes activated, leading to luminal thrombus formation (Steinhubl and Moliterno, 2005; Otsuka et al., 2016).

### Overall assessment of the AOP

#### Essentiality of KEs

Supporting evidence for KE essentiality are summarized in Supplementary Table SA1. Most KEs were rated as high because there is much clinical, animal model, and *in vitro* evidence demonstrating that blocking them would prevent or attenuate the downstream KEs. However, KE4 (Leukocyte recruitment/activation) was rated as moderate because there is only indirect evidence that Leukocyte recruitment/activation is necessary for endothelial dysfunction in the context of cigarette smoke-induced oxidative stress. Several studies indicate that cigarette smoke-induced oxidative stress promotes monocyte/macrophage recruitment to the vascular wall accompanied by vascular inflammation, endothelial damage and dysfunction (Orosz et al., 2007; Edirisinghe et al., 2008; Mao et al., 2021). Inhibition of oxidative stress suppresses cigarette smoke-induced monocyte/macrophage migration and adhesion to the endothelium, but there is a lack of direct evidence showing that inhibition of monocyte/macrophage activation, migration and adhesion, is followed by the suppression of endothelial injury and improved vascular function, under the cigarette smoke action (Orosz et al., 2007; Mao et al., 2021). According to the OECD guideline it corresponds to the moderate essentiality of KE.

KE7 (Increased platelet aggregation) was rated as low because there is no experimental evidence that blocking or attenuating platelet aggregation influences the downstream AO (Plaque progression). Cigarette smoke causes platelet activation and aggregation (Ichiki et al., 1996; Takajo et al., 2001). Smoking cessation, most likely by decreasing oxidative stress, can ameliorate the enhanced platelet aggregability in long-term smokers (Morita et al., 2005). Platelet aggregability is one of the strongest risk factors for atherosclerosis progression in smokers (Salonen and Salonen, 1990). In an animal model study, Huo and colleagues demonstrated that perfusion of activated platelets increased atherosclerotic lesions formation (Huo et al., 2003). Many studies demonstrate that platelet activation, adhesion, and aggregation at sites of vascular endothelial disruption are key events in pathogenesis of atherothrombosis, a condition which is characterized by atherosclerotic plaque disruption with superimposed thrombosis (Viles-Gonzalez et al., 2004; Steinhubl and Moliterno, 2005; Csordas and Bernhard, 2013; Martin-Ventura et al., 2017). However, there are no experimental studies demonstrating that ablation of smoking-induced platelet aggregation ameliorates atherosclerotic plaque progression. Thus, the essentiality of this KE is rated low.

### Biological plausibility of KERs

Supporting evidence for KERs biological plausibility are explained in detail in Supplementary Table SA2.

Most of the KERs from this AOP have a well-established mechanistic basis and there is much supporting evidence from human, animal model and *in vitro* studies. Therefore, we have rated the weight of most KERs as high (strong) with respect to biological plausibility.

However, the weight of KER (KE7=>AO) moderate, as scientific understanding of the mechanism of how smoking-induced platelet aggregation influences atherosclerotic plaque progression is not completely established (Salonen and Salonen, 1990; Huo et al., 2003). Many studies demonstrate that platelet activation, adhesion, aggregation, and activation of the coagulation cascade at sites of vascular endothelial disruption upon plaque rupture are key events in pathogenesis of atherothrombosis, which is characterized by atherosclerotic lesion disruption with superimposed thrombus formation (Viles-Gonzalez et al., 2004; Steinhubl and Moliterno, 2005; Csordas and Bernhard, 2013; Martin-Ventura et al., 2017). Moreover, there is some evidence that platelet activation takes place in the different phases of atherosclerosis; activated platelets are able to interact with endothelium and influence the development and progression of atherosclerotic plaque (Mustard and Packham, 1975; Sinzinger, 1986; Huo and Ley, 2004; Jørgensen, 2006; Gawaz et al., 2008; Wang and Tang, 2020). Several studies demonstrate that smoking-induced oxidative stress augments platelet aggregability which may contribute to atherothrombosis (Ichiki et al., 1996; Takajo et al., 2001). But there is a limited amount of experimental data confirming that smoking-induced platelet aggregation influences early atherosclerotic plaque formation and progression (Salonen and Salonen, 1990; Huo et al., 2003).

### Empirical support of KERs

An overview of supporting empirical evidence for the KERs is presented in Supplementary Table SA3. Selection criteria applied to this evidence were based on the OECD guidance and handbook for the development of the AOP.

The proposed AOP is a qualitative one. There is a good qualitative and quantitative understanding of how cigarette smoking-mediated oxidative stress affects lipid peroxidation, oxidized LDL uptake by macrophages, foam cell formation, increase in pro-inflammatory mediators, leukocyte recruitment and activation, NO depletion, endothelial dysfunction, and platelet aggregation on the cellular, tissue and organism level, lending strong support for these KERs (MIE =>KE1, KE1=>KE2, KE2=>AO, MIE =>KE3, KE3=>KE4, KE4=>KE6, MIE=>KE5, KE5=>KE6, KE6=>AO, KE5=>KE7, KE7=>AO). In addition, some of these studies indicate a dose-dependent relationship and causality for smoking-induced oxidative stress and associated atherosclerotic changes.

While cause and effect relationships are established for cigarette smoking-induced oxidative stress and platelet aggregation, the dose/time–response relationship is more difficult to define for KE7=>AO (Increased platelet aggregation leads to Plaque progression). There are several human and animal model studies that experimentally demonstrate the association of platelet aggregation and atherosclerosis progression (Salonen and Salonen, 1990; Huo et al., 2003). However, the exact and direct mechanism and causal relationship are unclear. Thus, the empirical support of this KER (KE7=>AO) is supposed to be low. Taking this together with weak essentiality of KE7 (KE7: Increase, platelet aggregation) and moderate biological plausibility of KE7=>AO (Increased platelet aggregation leads to Plaque progression) in the context of the AOP, the total weight of evidence for KE7 is believed to be low and we suppose that this KER (KE7=>AO) is indirect (see dashed line arrow in Figure 1).

### Pathways and molecular mechanisms

**FIGURE 2:**
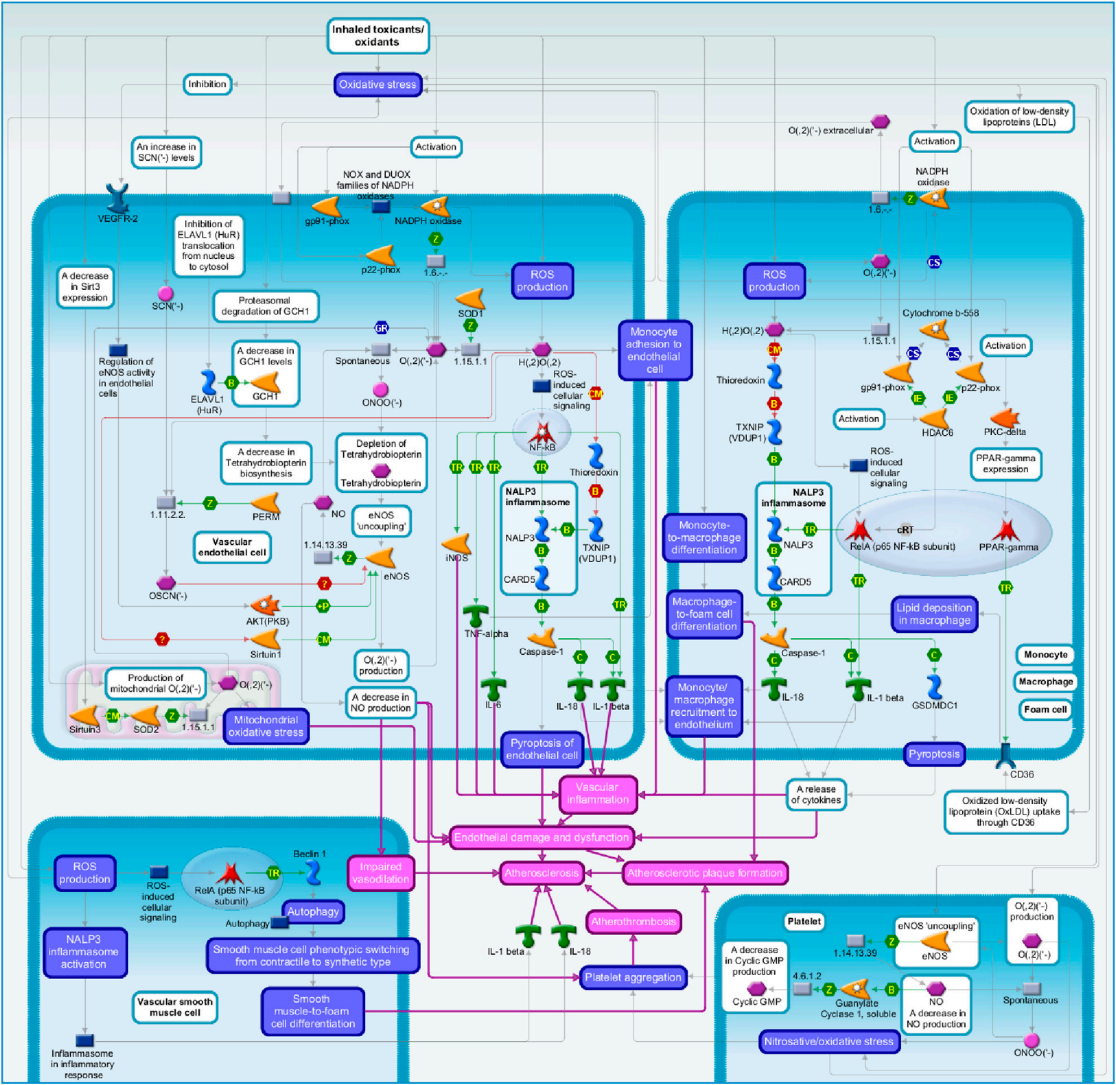
Summary of the signaling pathways: the role of oxidative stress induced by cigarette smoke in atherosclerosis.

A summary of putative signaling pathways is presented in Figure 2.

As mentioned above, oxidative stress was proposed as the main mechanism through which smoking promotes atherosclerosis formation (Ambrose and Barua, 2004; Messner and Bernhard, 2014; Siasos et al., 2014; Förstermann et al., 2017; El-Mahdy et al., 2020; Wang et al., 2021). Cigarette smoke-mediated oxidative stress induces the development and progression of atherosclerotic plaque via multiple molecular pathways which are summarized in this section.

Inhaled toxicants/oxidants, such as components of the gas/vapor or tar phase of cigarette smoke may be immediate sources of ROS (Ambrose and Barua, 2004) or may increase ROS production acting on vascular resident cells (endothelial cells) (Ambrose and Barua, 2004; Orosz et al., 2007; El-Mahdy et al., 2020; Wang et al., 2021), smooth muscle cells (Martin-Ventura et al., 2017; Wang et al., 2019; Yao et al., 2019; Wang et al., 2021) and vascular infiltrating cells (monocytes/macrophages) (Ambrose and Barua, 2004; El-Mahdy et al., 2020) and platelets (Takajo et al., 2001; Martin-Ventura et al., 2017) that produce ROS in response to cigarette smoke (Ambrose and Barua, 2004; Messner and Bernhard, 2014; Martin-Ventura et al., 2017; El-Mahdy et al., 2020; Wang et al., 2021).

In endothelial cells, cigarette smoke increases the levels of gp91-phox and p22-phox subunits of NADPH oxidase (Orosz et al., 2007; El-Mahdy et al., 2020), leading to ROS generation (Bedard and Krause, 2007; Orosz et al., 2007; El-Mahdy et al., 2020). Activated NADPH oxidase catalyzes the formation of O_2_^−^ (Orosz et al., 2007; Bedard and Krause, 2007), while superoxide dismutase, such as SOD1, catalyzes the conversion of O_2_^−^ to H_2_O_2_ (Orosz et al., 2007; Fukai and Ushio-Fukai, 2011). Moreover, cigarette smoke and its components induce ROS production in monocytes, macrophages and foam cells (Wu et al., 2018; Mehta and Dhawan, 2020; El-Mahdy et al., 2020; Mehta et al., 2020b; Mao et al., 2021). Cigarette smoke induces the activation and expression of gp91-phox and p22-phox in monocytes and macrophages, increasing NADPH oxidase-mediated O_2_^−^ production (El-Mahdy et al., 2020).

Cigarette smoke increases intracellular ROS levels at all stages of monocyte-to-macrophage-to-foam cell differentiation (Lugg et al., 2022). Cigarette smoke-induced oxidative stress induces lipid peroxidation significantly increasing ox-LDL levels (Scheffler et al., 1992; Heinecke et al., 1986; Frei et al., 1991; Morrow et al., 1995; Churg and Cherukupalli, 1993; Yamaguchi et al., 2005; Kunitomo et al., 2009; Arai, 2014; Messner and Bernhard, 2014). Moreover, in monocytes/macrophages, production of ROS is required for phosphorylation and activation of PKC-delta that then, via an unknown pathway, increases PPARγ expression (Feng et al., 2000; Dressman et al., 2003; Zhou et al., 2013). In turn, PPARγ upregulates the expression of CD36 (Feng et al., 2000; Dressman et al., 2003; Silverstein and Febbraio, 2009; Zhou et al., 2013), a scavenger receptor that mediates the recognition and uptake of ox-LDL (Febbraio et al., 2001; Silverstein and Febbraio, 2009). CD36 recognizes and internalizes ox-LDL, subsequently leading to lipid accumulation in macrophages (Liu et al., 2018). This results in the formation of foam cells containing lipids, ultimately promoting the progression of atherosclerotic plaques (Febbraio et al., 2001; Silverstein and Febbraio, 2009; Sakakura et al., 2013; Zhou et al., 2013; Feingold et al., 2000) and differentiate into lipid-laden foam cells, promoting atherosclerotic plaque development (Peluso et al., 2012; Yu et al., 2013; Chistiakov et al., 2017; Orekhov, 2018).

Cigarette smoke components increase ROS production in vascular smooth muscle cells. In turn, ROS activate RelA (p65 NF-κB subunit) that increases the expression of Beclin 1 (Wang et al., 2019), a trigger protein in autophagy (Cao and Klionsky, 2007; Martinet and De Meyer, 2009; Wang et al., 2019). Autophagy further contributes to the phenotypic switching of smooth muscle cells from the contractile to the synthetic type (Wang et al., 2019), which enhances the cell abilities to migrate and proliferate (Xie et al., 2011; Siasos et al., 2014; Wang et al., 2019; Liu et al., 2021). Upon migration to the intima, vascular smooth muscle cells can trans-differentiate into lipid-laden foam cells, leading to atherosclerotic plaque formation and atherogenesis (Wolfbauer et al., 1986; Wang et al., 2019; Liu et al., 2021).

Cigarette smoke-induced oxidative stress promotes proinflammatory alterations in the vascular endothelium (Ambrose and Barua, 2004; Orosz et al., 2007; Messner and Bernhard, 2014). H_2_O_2_, produced in endothelial cells, leads to the activation of NF-κB, which, probably, contributes to the expression of proinflammatory factors, such as iNOS (Orosz et al., 2007; Morgan and Liu, 2011) and cytokines TNFα, IL-6, and IL-1β (Brand et al., 1997; Orosz et al., 2007; Checa and Aran, 2020). H_2_O_2_, at least in part via the production of cytokines such as TNFα, also induces monocyte adhesion to endothelial cells (Orosz et al., 2007).

Moreover, ROS activate NALP3 inflammasome in endothelial cells (Schroder and Tschopp, 2010; Latz, 2010; Wu et al., 2018; Qian et al., 2021) and H_2_O_2_ most likely via NF-kB and/or TXNIP (VDUP1)-dependent pathways activates NALP3 (Zhou et al., 2010; Tschopp and Schroder, 2010; Schroder and Tschopp, 2010; Yin et al., 2017). H_2_O_2_ blocks Thioredoxin association with TXNIP (VDUP1), causing TXNIP (VDUP1) interaction with NALP3 (Zhou et al., 2010). Within the inflammasome, activated NALP3 binds to the adapter protein CARD5 that, in turn, associates with and activates Caspase-1 (Schroder and Tschopp, 2010; Latz, 2010; Wu et al., 2018). Activated Caspase-1 triggers endothelial cell pyroptosis, a cell death mechanism that results in cell lysis (Qian et al., 2021). Also, activated Caspase-1 cleaves IL-18 and IL-1β, thereby processing mature inflammatory cytokines IL-18 and IL-1β. Endothelial cells lysis leads to cytokines release promoting vascular inflammation and monocyte/macrophage migration and recruitment to early atherosclerotic plaques (Miao et al., 2011; Wu et al., 2018; Qian et al., 2021).

In monocytes, macrophages and foam cells, oxidative stress also activates NALP3 inflammasome via the H_2_O_2_/Thioredoxin/TXNIP (VDUP1) pathway (Zhou et al., 2010; Mehta and Dhawan, 2020; Mehta et al., 2020a; Mehta et al., 2020b; Mao et al., 2021). NALP3 via CARD5 activates Caspase-1 signaling leading to IL-18 and IL-1β processing and activation (Mehta and Dhawan, 2020; Mehta et al., 2020a; Mehta et al., 2020b; Mao et al., 2021). Caspase-1 also cleaves GSDMDC1 which forms pores in the cell membrane and stimulates cell pyroptosis (Mao et al., 2021; Qian et al., 2021). Then, the inflammatory cytokines along with other alarmins are released through cell membrane pores or after membrane lysis (Qian et al., 2021). In addition, cigarette smoke components induce macrophage pyroptosis through the activation of HDAC6. Deacetylase HDAC6 reduces the acetylation level of RelA (p65 NF-κB subunit), thus enhancing RelA (p65 NF-κB subunit) nuclear translocation. In the nucleus, RelA (p65 NF-κB subunit) upregulates the expression of NALP3, leading to the activation of NALP3 inflammasome (Xu et al., 2021). In macrophages, HDAC6 can also upregulate the expression and activity of p22-phox and gp91-phox, two subunits of Cytochrome b-558 that, in turn, is a part of NADPH oxidase (Bedard and Krause, 2007; Youn et al., 2016). NADPH oxidase produces O_2_^−^ that then rapidly dismutates to H_2_O_2_ (Bedard and Krause, 2007). At least in part, HDAC6-induced NADPH oxidase/H_2_O_2_ signaling leads to the activation of RelA (p65 NF-κB subunit) and production of inflammatory cytokines such as IL-1β(Morgan and Liu, 2011; Zhang et al., 2016; Youn et al., 2016). In vascular smooth muscle cells, ROS production also leads to the activation of NALP3 inflammasome and release of mature IL-1β and IL-18 (Grebe et al., 2018; Yao et al., 2019).

Oxidative stress-induced increase in proinflammatory factors and monocyte/macrophage recruitment to the vascular wall leads to vascular inflammation. Moreover, endothelial cell, macrophage and foam cell pyroptosis increases inflammation and necrotic core formation in advanced atherosclerosis, promoting endothelial damage and dysfunction, which results in the progression of atherosclerosis (Ambrose and Barua, 2004; Orosz et al., 2007; Messner and Bernhard, 2014; Edirisinghe et al., 2008; Mao et al., 2021; Qian et al., 2021).

Also, in endothelial cells, increased oxidative stress promotes oxidation and depletion of Tetrahydrobiopterin (Crabtree and Channon, 2011; El-Mahdy et al., 2020). Tetrahydrobiopterin plays a crucial role in regulating eNOS activity. When monomeric eNOS forms a stable dimer through heme and Tetrahydrobiopterin, eNOS becomes biologically active (Wang et al., 2021). Active eNOS catalyzes NO biosynthesis (Alderton et al., 2001). Depletion of Tetrahydrobiopterin leads to eNOS ‘uncoupling’, a condition in which eNOS produces O_2_^−^ rather than NO leading to decrease in NO levels (Crabtree and Channon, 2011; El-Mahdy et al., 2020). Notably, the reaction between NO and O_2_^−^ results in ONOO^−^ generation; ONOO^−^ interaction with Tetrahydrobiopterin is a more probable mechanism for Tetrahydrobiopterin oxidation (Crabtree and Channon, 2011; Siasos et al., 2014). Extracellular O_2_^−^ produced by monocytes/macrophages can penetrate into the adjacent vascular endothelial cells and increase endothelial intracellular O_2_^−^ levels, facilitating ROS-dependent eNOS ‘uncoupling’ (El-Mahdy et al., 2020). Both NADPH oxidase and eNOS-derived ROS can feedback on each other resulting in a vicious cycle of monocyte/macrophage and endothelial-mediated oxidant stress that causes vascular dysfunction in atherosclerosis (El-Mahdy et al., 2020). In endothelial cells, cigarette smoke also induces proteasomal degradation of GCH1, a rate-limiting enzyme in Tetrahydrobiopterin biosynthesis (Abdelghany et al., 2018). Moreover, cigarette smoke components inhibit ELAVL1 (HuR) translocation from the nucleus to the cytosol and suppress ELAVL1 (HuR)-mediated stability of GCH1 mRNA (Li et al., 2018). A decrease in GCH1 levels leads to a decrease in Tetrahydrobiopterin biosynthesis followed by eNOS ‘uncoupling’ (Abdelghany et al., 2018; Li et al., 2018). The impairment of endothelial Tetrahydrobiopterin synthesis and eNOS ‘uncoupling’ contribute to an increase in O_2_^−^ production and a decrease in NO production (Alp et al., 2004; Crabtree and Channon, 2011; Li et al., 2018; Wang et al., 2021).

In addition, cigarette smoke increases the levels of SCN- in endothelial cells (Morgan et al., 2011; Wang et al., 2021). Also, cigarette smoke-induced oxidative stress increases the availability of H_2_O_2_. PERM uses H_2_O_2_ to catalyze the conversion of SCN^−^ to OSCN^−^ (Talib et al., 2014; Wang et al., 2021). In turn, OSCN^−^ disrupts eNOS dimer structure and thus inhibits eNOS activity, leading to decreased eNOS-mediated NO production (KE5: NO depletion) (Talib et al., 2014).

Also, cigarette smoke-induced ROS inhibit the expression and activity of VEGFR-2 in endothelial cells (Edirisinghe et al., 2008; Edirisinghe and Rahman, 2010). VEGFR-2 inactivation attenuates VEGFR-2-induced AKT (PKB) phosphorylation and activity, which, in turn, attenuates AKT (PKB)-induced eNOS phosphorylation and activity (Jin et al., 2003; Edirisinghe et al., 2008; Edirisinghe and Rahman, 2010; El-Mahdy et al., 2020; Wang et al., 2021).

In endothelial cells, cigarette smoke induced H_2_O_2_ reduces Sirtuin1 levels and decreases its deacetylase activity, leading to increased eNOS acetylation and reduced NO production (Arunachalam et al., 2010; Edirisinghe and Rahman, 2010; Wang et al., 2021). Moreover, cigarette smoke decreases the expression of the mitochondrial deacetylase Sirtuin3 in endothelial cells (Dikalov et al., 2019). Under normal conditions, Sirtuin3 deacetylates a key mitochondrial antioxidant SOD2 to maintain its activity. Superoxide scavenger SOD2 reduces O_2_^−^ accumulation and protects against oxidative stress (Sun et al., 2018). Attenuation of Sirtuin3-mediated deacetylation increases the mitochondrial oxidative stress and leads to the mitochondrial O_2_^−^ accumulation. O_2_^−^ overproduction contributes to eNOS ‘uncoupling’ leading to decrease in NO production (Dikalov et al., 2019; Wang et al., 2021).

As a result, decreased NO production subsequently leads to endothelial dysfunction promoting atherosclerosis (Gimbrone and García-Cardeña, 2016; Malekmohammad et al., 2019; El-Mahdy et al., 2020; Wang et al., 2021).

Cigarette smoke-induced oxidative stress also promotes platelet aggregation (Takajo et al., 2001). Oxidative stress, which is accompanied by enhanced intraplatelet O_2_^−^ levels, leads to eNOS ‘uncoupling’ in platelets, that results in a decreased NO production and increased O_2_^−^ (Takajo et al., 2001; Förstermann and Sessa, 2012; Alexandru et al., 2010; Gawrys et al., 2020). Moreover, spontaneous reaction between NO and O_2_^−^ leads to ONOO^−^ synthesis resulting in nitrosative/oxidative stress in platelets (Takajo et al., 2001; Alexandru et al., 2010; Förstermann and Sessa, 2012; Gawrys et al., 2020). NO depletion leads to reduced activation of Guanylate Cyclase 1 soluble and a decrease in Cyclic GMP levels (Dangel et al., 2010; Radziwon-Balicka et al., 2017; Makhoul et al., 2018). Subsequently, both nitrosative/oxidative stress and a decrease in Cyclic GMP levels can lead to platelet aggregation (Takajo et al., 2001; Dangel et al., 2010; Alexandru et al., 2010; Makhoul et al., 2018). Increased platelet aggregation may contribute to atherosclerotic plaque formation (Salonen and Salonen, 1990; Huo et al., 2003).

### Domain of applicability

#### Taxonomic applicability

The evidence presented in this study in support of the proposed AOP is derived from human, rat, and mouse biological systems. Also, there was a small amount of data included that was generated using non-human primate (Heinecke et al., 1986) and bovine (Jaimes et al., 2004) cells*. In vitro* and *in vivo* studies in these test systems have been used to clarify the mechanisms of smoking-induced oxidative stress in plaque formation. Clinical data was obtained from patients with atherosclerosis, healthy subjects, smokers or non-smokers using blood or plasma samples. *In vitro* human data were obtained using human cell cultures. Animal *in vitro* and *in vivo* data were obtained using genetically modified animal model systems (ApoE^−/−^ mice), blood or tissue samples, and *in vitro* animal cell cultures.

In summary, collected evidence suggests that data obtained from human and animal studies are consistent and the majority of KEs are conserved and relevant regardless of species used for the test system.

### Life stage and sex applicability

Smoking-induced atherosclerosis and related biological mechanisms in humans were studied predominantly in middle-aged adults. No child studies were observed in the context of AOP. Study groups included generally healthy smokers and non- or never-smokers, and patients with cardiovascular manifestations.

Most smoking-related clinical studies used male subjects. Several studies included mixed male-female groups (Heitzer et al., 1996; Valkonen and Kuusi, 1998; Valkonen and Kuusi, 2000; Papamichael et al., 2004; Solak et al., 2005), and one study was performed in females only (Bergmann et al., 1998). In terms of smoking status, female were more dominantly mild smokers, whereas males were more dominantly observed to be heavy smokers (Solak et al., 2005). However, the available clinical evidence in support of the AOP suggests that there is no remarkable gender difference in level of smoking.

### Application of the AOP

Atherosclerosis and its clinical manifestations, such as ischemic heart disease, stroke, and peripheral arterial disease, are the leading causes of vascular injury-related death in the world. But the etiology of atherosclerosis and exact mechanisms involved in atherosclerotic plaque formation are not fully understood to date. Smoking is one of the major preventable risk factors for atherosclerosis and most types of cardiovascular disease. The proposed AOP provides a mechanistic model of how oxidative stress caused by inhaled toxicants/oxidants from cigarette smoke can lead to the formation of atherosclerosis. Assessment of described biological endpoints also provides a mechanistic basis of smoking-induced atherosclerosis. Moreover, the AOP described here may help to evaluate novel targets for early diagnostics and more effective treatment options.

### Biomarkers

This AOP can be used as a source for relevant biomarkers of effect of cigarette smoking and/or oxidative stress, with the aim of developing reliable preclinical markers that are indicative of the atherosclerosis plaque formation.

Biomarkers and corresponding assays which were used to measure the MIE, each KE and AO of this AOP are summarized in Supplementary Table SA4.

Based on the cumulative weight of evidence for KEs for this AOP and number of studies which measure the selected biomarkers in context of smoking and oxidative stress, we propose that these biomarkers may have clinical significance to predict or indicate the risk of atherosclerosis formation in smokers. Thus, increased markers of oxidative stress, increased lipid peroxides and thiobarbituric acid reactive substances (TBARS), increased expression levels of macrophage surface markers, increased NLRP3 inflammasome assembly markers and vascular inflammatory mediators, markers of NO depletion and endothelial cell dysfunction may be used to develop relevant clinical tests for atherosclerosis assessment in smokers. Increased platelet aggregability has low essentiality and biological plausibility for atherosclerotic plaque formation, but it could serve as relevant marker of thrombosis (as acute complication of atherosclerosis).

## Conclusion

In conclusion, in this manuscript we propose the AOP framework which represents a mechanistic relationship between cigarette smoke-mediated oxidative stress and atherosclerotic plaque formation. Molecular mechanisms underlying the pathogenesis of atherosclerosis have been extensively investigated during the past 30 years, however there are still many gaps of knowledge. The overall weight of collected evidence supporting KEs and KERs in this AOP is strong. Obtaining measures of AOPs in the clinic presents significant challenges. KE in AOPs often involve intricate molecular and cellular interactions that can be difficult to measure directly, offer few validated biomarkers and those present often these often overlap with comorbidities. *In vitro* assays and new approach methodologies (NAMs) may offer promising tools to address these issues. These alternatives enable controlled, mechanistic investigations of cellular responses to stressors, facilitating as proxies to bridge the gap between clinically measurable key events to enhance the predictive power of AOP frameworks and improve risk assessment strategies.

## Abbreviations

AKT (PKB), AKT serine/threonine kinase (protein kinase B); AO, adverse outcome; AOP, adverse outcome pathway; CARD5, PYD and CARD domain containing; CD36, CD36 molecule; Cyclic GMP, Cyclic guanosine monophosphate; DNA, Deoxyribonucleic acid; ELAVL1 (HuR), ELAV like RNA binding protein 1; eNOS, Endothelial nitric oxide synthase; GCH1, GTP cyclohydrolase 1; gp91-phox, cytochrome b-245 beta chain; GSDMDC1, asdermin D; HDAC6, histone deacetylase 6; IL-1 β, interleukin 1 beta; IL-18, interleukin 18; IL-6, interleukin 6; iNOS, inducible nitric oxide synthase; KE, key event; LDL, low density lipoproteins; MIE, molecular initiating event; MMPs, matrix metalloproteinase; NADPH oxidase, nicotinamide adenine dinucleotide phosphate oxidase; NALP3, NLR family pyrin domain containing 3; NF-kB, nuclear factor kappa-light-chain-enhancer of activated B cells; NO, nitric oxide; Organisation for Economic Co-operation and Development (OECD); ox-LDL, oxidized-low density lipoproteins; p22-phox, cytochrome b-245 alpha chain, PERM, myeloperoxidase; PIT, pathological intimal thickening; PKC-delta, protein kinase C delta; PPAR-gamma peroxisome proliferator activated receptor gamma, RelA (p65 NF-kB subunit), RELA proto-oncogene, NF-kB subunit; RNS, reactive nitrogen species; ROS, reactive oxygen species; SCN(’-), Thiocyanate; SOD1, superoxide dismutase 1; TBARS, thiobarbituric acid reactive substances; TCFA, thin cap fibroatheroma; TNF-alpha, tumor necrosis factor; TXNIP (VDUP1), thioredoxin interacting protein; VEGFR-2, kinase insert domain receptor.
