# Supplementary material for: An adverse outcome pathway for cigarette smoke-mediated oxidative stress in plaque formation
Source: Front Toxicol. 2025 Jun 30;7:1554747. doi: 10.3389/ftox.2025.1554747 (PMC12256450; doi:10.3389/ftox.2025.1554747)
Supplement: Supplementary file 1 [file Table1.docx]

# The Adverse Outcome Pathway for the Cigarette Smoke-mediated Oxidative Stress in Atherosclerosis

## Appendix A. Supplementary data

### Table A.1. Supporting Essentiality of KEs

| **Support for Essentiality of KEs** | ***Defining Question*** | ***High (Strong)*** | ***Moderate*** | ***Low (Weak)*** |
| --- | --- | --- | --- | --- |
|  | *Are downstream KEs and/or the AO prevented if an upstream KE is blocked?* | *Direct evidence from specifically designed experimental studies illustrating essentiality for at least one of the important KEs* | *Indirect evidence that sufficient modification of an expected modulating factor attenuates or augments a KE* | *No or contradictory experimental evidence of the essentiality of any of the KEs.* |
| Stressor: Cigarette smoke-derived oxidants and reactive oxygen species | **High** | - Many experimental and clinical data demonstrate that cigarette smoke exposure increases oxidative stress as a potential mechanism for initiating and progression of atherosclerosis (Ambrose & Barua, 2004; Howard et al., 1998; Kunitomo et al., 2009; Li et al., 2018; Mao et al., 2021; Mehta & Dhawan, 2020; Salonen & Salonen, 1990; Scheffler et al., 1992; Talib et al., 2014; Wang et al., 2019; Wu et al., 2018). | | |
| MIE: Oxidative Stress | **High** | - Oxidative stress is a result of cigarette smoke-derived oxidants (gas and tar phase of cigarette smoke) and cigarette smoke-induced production of reactive oxygen species (ROS) by vascular resident (endothelial cells, smooth muscle cells) and infiltrating cells (monocytes/macrophages) (Ambrose & Barua, 2004; El-Mahdy et al., 2020; Orosz et al., 2007; Takajo et al., 2001; Wang et al., 2019; Yamaguchi et al., 2007). - Oxidative stress has a pivotal role in the progression of atherosclerosis (Malekmohammad et al., 2019). - Suppression of oxidative stress by antioxidants markedly improved endothelial dysfunction (an early stage of atherosclerosis) in chronic smokers (Heitzer, Just, et al., 1996; Papamichael et al., 2004; Valkonen & Kuusi, 1998), and reduced the smoke-induced lipid peroxidation (Frei et al., 1991; Valkonen & Kuusi, 2000). - In animal in vitro study inhibitors of oxidative stress (such as apocynin and catalase) prevented proinflammatory alterations and vascular dysfunction induced by cigarette smoke extract (Orosz et al., 2007). | | |
| KE1: Increased oxidized LDL uptake by macrophages | **High** | - Macrophages have a key role in the early development of atherosclerosis. After differentiation from peripheral blood monocytes, intimal macrophages incorporate oxidized LDL and differentiate into foam cells (Arai, 2014; Orekhov, 2018). - Human and animal studies demonstrated that cigarette smoking facilitates oxidative modification of LDL and increase of oxidazed LDL uptake by macrophages and thereby contributes to atherosclerosis development (Heitzer, Ylä-Herttuala, et al., 1996; Kunitomo et al., 2009; Rom & Aviram, 2016; Scheffler et al., 1992; Valkonen & Kuusi, 1998). | | |
| KE2: Foam cell formation | **High** | - It is well-established that differentiation in monocytes, macrophages and foam cells are the key stages in atherosclerotic plaque development (Chistiakov et al., 2017; Yu et al., 2013), (Orekhov, 2018). - Cigarette smoke condensate (CSC) and cigarette smoke components could induce differentiation of human monocytes into macrophages and foam cells in a ROS-dependent manner (Mehta & Dhawan, 2020; Zhou et al., 2013). - Cigarette smoke components increased oxLDL-induced lipid accumulation and foam cell formation, which was prevented by siRNA to CD36, which is a major macrophage receptor for proatherogenic oxidized LDLs (Silverstein & Febbraio, 2009; Zhou et al., 2013). | | |
| KE3: Increased pro-inflammatory mediators | **High** | - The inflammatory response is an essential component in the initiation and evolution of atherosclerosis (Ambrose & Barua, 2004; Tousoulis et al., 2016). - Several studies experimentally demonstrated that cigarette smoke-induced oxidative stress promoted increased secretion of proinflammatory mediators (such as IL-1 beta, IL-18, TNF-alpha, and others) from endothelial cells, vascular smooth muscle cells, and macrophages leading to proinflammatory alterations in vascular phenotype (Mao et al., 2021; Mehta, Vijayvergiya, et al., 2020; Orosz et al., 2007; Wu et al., 2018). - Oxidative stress inhibitors and silencing of NLRP3 inflammasome prevented pyroptosis and secretion of inflammatory cytokines in endothelial cells, vascular smooth muscle cells, and macrophages (Mao et al., 2021; Wu et al., 2018; Yao et al., 2019). - There is a large body of evidence connecting the NLRP3 inflammasome, pyroptosis and atherosclerosis (Grebe et al., 2018; Qian et al., 2021). | | |
| KE4: Leukocyte recruitment/ activation | **Moderate** | - Several studies indicate that cigarette smoke-induced oxidative stress promoted proinflammatory factors secretion and monocyte/ macrophage recruitment to the vascular wall leading to vascular inflammation, endothelial damage and dysfunction (Bergmann et al., 1998; Edirisinghe et al., 2008; Mao et al., 2021; Orosz et al., 2007). - Inhibitors of oxidative stress suppressed cigarette smoke-induced monocyte adhesion to the endothelium (Orosz et al., 2007). - Inhibitors of ROS decreased cigarette smoke components-induced macrophage migration and subsequent endothelial injury (Mao et al., 2021). | | |
| KE5: NO depletion | **High** | - Many studies demonstrated that oxidative stress induced by various toxicants (including cigarette smoke) causes NO depletion (Abdelghany et al., 2018; Barua et al., 2003; El-Mahdy et al., 2020). - Cigarette smoking-induced oxidative stress causes decrease of Tetrahydrobiopterin bioavailability (a required cofactor for NO synthesis by endothelial nitric oxide synthase (eNOS)) leading to eNOS uncoupling. Dysfunction of eNOS promotes generation of superoxide instead of NO leading to NO depletion (Abdelghany et al., 2018; El-Mahdy et al., 2020; Heitzer et al., 2000). - Reversal of eNOS uncoupling is achieved by supplementation of tetrahydrobiopterin, which restores NO production and improves cigarette smoke-induced endothelial dysfunction (Alp et al., 2004; Heitzer et al., 2000; Li et al., 2018). | | |
| KE6: Endothelial cell dysfunction | **High** | - Multiple studies demonstrate that smoking-induced oxidative stress is associated with endothelial dysfunction, which is an early stage of atherosclerosis (Edirisinghe et al., 2008; Heitzer, Just, et al., 1996; Li et al., 2018; Papamichael et al., 2004). - Supplementation of tetrahydrobiopterin, which restores NO production, improves cigarette smoke-induced endothelial dysfunction (Alp et al., 2004; Heitzer et al., 2000; Li et al., 2018). - Inhibition of NAD(P)H oxidase (which is a source of vascular superoxide production) by apocynin improves smoking-induced endothelial dysfunction in rats in vivo (Orosz et al., 2007). | | |
| KE7: Increase, platelet aggregation | **Low** | - Many studies demonstrate that platelet activation, adhesion, and aggregation at sites of vascular endothelial disruption are key events in pathogenesis of atherothrombosis (Csordas & Bernhard, 2013; Martin-Ventura et al., 2017; Steinhubl & Moliterno, 2005; Viles-Gonzalez et al., 2004). - Cigarette smoke causes platelet activation and aggregation (Ichiki et al., 1996; Takajo et al., 2001). Smoking cessation can ameliorate the enhanced platelet aggregability and intraplatelet redox imbalance in long-term smokers, possibly by decreasing oxidative stress (Morita et al., 2005). - The role of smoking-induced platelet aggregation in atherosclerotic plaque progression is not fully understood. Platelet aggregability along with pack-years of smoking was one of the strongest predictors of atherosclerosis progression (Salonen & Salonen, 1990). - In an animal model study was demonstrated that perfusion of activated platelets increased atherosclerotic lesions formation in Apoe–/– mice (Huo et al., 2003). | | |

### Table A.2. Supporting Biological Plausibility of KERs

| **1. Support for Biological Plausibility of KERS** | ***Defining Question*** | ***High (Strong)*** | ***Moderate*** | ***Low (Weak)*** |
| --- | --- | --- | --- | --- |
|  | *a) Is there a mechanistic relationship between KEup and KEdown consistent with established biological knowledge?* | *Extensive understanding of the KER based on extensive previous documentation and broad acceptance.* | *KER is plausible based on analogy to accepted biological relationships, but scientific understanding is incomplete* | *Empirical support for association between KEs, but the structural or functional relationship between them is not understood* |
| MIE =>KE1 (Relashionship NEW): Oxidative Stress (Event 1392) leads to Increased oxidized LDL uptake by macrophages (Event NEW) | **High** | - Many studies demonstrated that cigarette smoke rapidly induces lipid peroxidation mediated by active oxygen species (Churg & Cherukupalli, 1993; Frei et al., 1991; Morrow et al., 1995; Yamaguchi et al., 2005). - Oxidative modification of low-density lipoprotein (LDL) (or other lipoproteins) is important and possibly obligatory in the pathogenesis of the atherosclerotic lesion (Feingold et al., 2000; Orekhov, 2018; Steinberg et al., 1989; Witztum, 1994). - Oxidative stress plays an important role in oxidation of LDL-cholesterol, which is thought to be more important that native-LDL in atherogenesis (Kattoor et al., 2017; Witztum, 1994). - Oxidative modification of LDL leads to enhanced uptake by macrophages (Arai, 2014; Heinecke et al., 1986; Peluso et al., 2012) | | |
| KE1=>KE2 (Relashionship NEW): Increased oxidized LDL uptake by macrophages (Event New) leads to Foam cell formation (Event 1442) | **High** | - It is well-documented that formation of macrophage foam cells in the intima is a major hallmark of early stage atherosclerotic lesions. Uncontrolled uptake of oxidized LDL, excessive cholesterol esterification and/or impaired cholesterol release result in accumulation of cholesterol ester stored as cytoplasmic lipid droplets and subsequently trigger the formation of foam cells (Feingold et al., 2000; Orekhov, 2018; Yu et al., 2013). - Macrophages serve as the main source of foam cells after they penetrate the endothelial barrier and accumulate in the arterial intima media (Chistiakov et al., 2017). - Human macrophages expressing scavenger receptors take up oxidized-low density lipoprotein (ox-LDL) and differentiate into lipid-laden foam cells (Mehta & Dhawan, 2020; Zhou et al., 2013). | | |
| KE2=>AO (Relationship 1594): Foam cell formation (Event 1442) leads to Plaque progression (Event 1443) | **High** | - It is well-established that differentiation in monocytes, macrophages and foam cells are the key stages in atherosclerotic plaque development (Chistiakov et al., 2017; Orekhov, 2018; Yu et al., 2013). - Cigarette smoke increases intracellular ROS levels at all stages of monocyte-to-macrophage-to-foam cell differentiation promoting initiation and progression of atherosclerosis (Mehta & Dhawan, 2020). - Cigarette smoke components increased CD36 expression in human THP1 macrophages resulting in resulting in macrophage activation and foam cell formation. In vivo Cigarette smoke components exacerbates atherosclerotic lesion formation through upregulation of CD36 (Feingold et al., 2000; Zhou et al., 2013). | | |
| MIE =>KE3 (Relashionship NEW): Oxidative Stress (Event 1392) leads to Increased pro-inflammatory mediators (KE 1493) | **High** | - There is large body of evidence that oxidative stress promotes proinflammatory alterations in the vascular endothelium associated with atherosclerosis (Checa & Aran, 2020; Mury et al., 2018; Spagnoli et al., 2007; Yokoyama, 2004). - Several studies reported that cigarette smoke components induce NADPH oxidase/ROS-dependent production of proinflammatory mediators (such as TNF-alpha, IL-6, and IL-1 beta, and IL-18) in the vascular endothelium associated with atherosclerosis (Ambrose & Barua, 2004; Messner & Bernhard, 2014; Orosz et al., 2007; Wu et al., 2018; Yao et al., 2019). | | |
| KE3=>KE4 (Relashionship 1777): Increased pro-inflammatory mediators (KE 1493) leads to Leukocyte recruitment/ activation (Event 1494) | **High** | - Inflammatory response is an essential component in the initiation and evolution of atherosclerosis (Ambrose & Barua, 2004; Tousoulis et al., 2016). - Proinflammatory factors promote monocyte recruitment to the vascular wall, maturation into macrophages and increasing vascular inflammation (Ambrose & Barua, 2004; Messner & Bernhard, 2014; Orosz et al., 2007; Qian et al., 2021; Sakakura et al., 2013). - Several studies indicate that cigarette smoke-induced oxidative stress promoted proinflammatory factors secretion and monocyte/ macrophage recruitment to the vascular wall leading to vascular inflammation and endothelial damage (Edirisinghe et al., 2008; Mao et al., 2021; Orosz et al., 2007). | | |
| KE4=>KE6 (Relashionship NEW): Leukocyte recruitment/ activation (Event 1494) leads to Endothelial cell dysfunction (Event 1913) | **High** | - Endothelial cell dysfunction is a complex pathogenic sequence, initially involving the selective recruitment of circulating monocytes from the blood into the intima, where they differentiate into macrophages and internalize modified lipoproteins to become foam cells (Gimbrone & García-Cardeña, 2016; Sakakura et al., 2013). - Several studies indicate that smoking (or cigarette smoke components) may accelerate atherosclerosis in part by promoting macrophage pyroptosis and endothelial damage and dysfunction (Edirisinghe et al., 2008; Mao et al., 2021; Messner & Bernhard, 2014; Orosz et al., 2007). | | |
| MIE=>KE5 (Relashionship NEW): Oxidative Stress (Event 1392) leads to NO depletion (Event 933) | **High** | - Many studies have provided compelling evidence demonstrating the role of vascular oxidative stress and NO in atherosclerosis (Alp et al., 2004; Crabtree & Channon, 2011; Forgione & Loscalzo, 2000; Förstermann et al., 2017). - Multiple studies reported that cigarette smoking-induced oxidative stress reduces bioavailability of tetrahydrobiopterin (a required cofactor for NO synthesis by endothelial nitric oxide synthase (eNOS)) and has been demonstrated to cause an uncoupling of eNOS thereby generating superoxide instead of NO leading to NO depletion (Abdelghany et al., 2018; Barua et al., 2003; El-Mahdy et al., 2020; Grassi et al., 2010; Heitzer et al., 2000; Jaimes et al., 2004; Wang et al., 2021). | | |
| KE5=>KE6 (Relashionship NEW): NO depletion (Event 933) leads to Endothelial cell dysfunction (Event 1913) | **High** | - It is well-known that nitric oxide (NO) plays a major role in the regulation of vascular tone, structure, and function, and reduced NO bioavailability directly contributes to development of vascular endothelial dysfunction and could be considered the first step in the pathogenesis of atherosclerosis (El-Mahdy et al., 2020; Grassi et al., 2010; Puranik & Celermajer, 2003; Sukhovershin et al., 2015). - Many human and animal studies demonstrate that cigarette smoking-induced oxidative leads to NO depletion and thereby promotes endothelial cell dysfunction (Abdelghany et al., 2018; Ambrose & Barua, 2004; Barua et al., 2003; El-Mahdy et al., 2020; Grassi et al., 2010; Heitzer et al., 2000; Heitzer, Just, et al., 1996; Jaimes et al., 2004; Puranik & Celermajer, 2003). | | |
| KE6=>AO (Relashionship NEW): Endothelial cell dysfunction (Event 1913) leads to Plaque progression (Event 1443) | **High** | - It is well-established that the endothelium plays a crucial role in the process of atherosclerotic disease by its regulatory functions on the vasculature (Gimbrone & García-Cardeña, 2016; Sakakura et al., 2013; Schächinger & Zeiher, 2000). - Many studies demonstrated that smoking-induced oxidative stress induced endothelial cell injury and endothelial dysfunction which lay in basis of aterosclerotic plaque development and progression (Chen et al., 2018; Messner & Bernhard, 2014; Münzel et al., 2020; Puranik & Celermajer, 2003; Schächinger & Zeiher, 2000; Siasos et al., 2014; Sukhovershin et al., 2015; Wang et al., 2021). | | |
| KE5=>KE7 (Relashionship NEW): NO depletion (Event 933) leads to Increase, platelet aggregation (Event 1914) | **High** | - It is well-established that nitric oxide (NO) signaling inhibits platelet aggregation (Dangel et al., 2010; Makhoul et al., 2018; Radziwon-Balicka et al., 2017). - Smoking impairs platelet-derived nitric oxide (PDNO) bioactivity and augments platelet aggregability (Ichiki et al., 1996; Takajo et al., 2001). - Smoking cessation can ameliorate the enhanced platelet aggregability and intraplatelet redox imbalance in long-term smokers, possibly by decreasing oxidative stress (Morita et al., 2005). | | |
| KE7=>AO (Relashionship NEW): Increase, platelet aggregation (Event 1914) leads to Plaque progression (Event 1443) | **Moderate** | - Many studies demontrate that platelet activation, adhesion, aggregation and activation of the coagulation cascade at sites of vascular endothelial disruption upon plaque rupture are key events in pathogenesis of atherothrombosis (Csordas & Bernhard, 2013; Martin-Ventura et al., 2017; Steinhubl & Moliterno, 2005; Viles-Gonzalez et al., 2004). - There are many evidence that platelet activation can be seen in the different phases of atherosclerosis, activated platelets are able to interact with endothelium and influence the development and progression of atherosclerotic plauque (Gawaz et al., 2008; Huo & Ley, 2004; Wang & Tang, 2020). - Several studies demonstrate that smoking-induced oxidative stress augments platelet aggregability which may contribute in atherothrombosis (Ichiki et al., 1996; Takajo et al., 2001). - There are small data about how smoking-induced platelet aggregation influences atherosclerotic plaque progression (Salonen & Salonen, 1990). | | |

### Table A.3. Supporting empirical evidence for the KERs.

| **Empirical Support for KERs** | ***Defining Question*** | ***High (Strong)*** | ***Moderate*** | ***Low (Weak)*** |
| --- | --- | --- | --- | --- |
|  | *Does empirical evidence support that a change in KEup leads to an appropriate change in KEdown? Does KEup occur at lower doses and earlier time points than KE down and is the incidence of KEup > than that for KEdown? Inconsistencies?* | *Multiple studies showing dependent change in both events following exposure to a wide range of specific stressors. No or few critical data gaps or conflicting data* | *Demonstrated dependent change in both events following exposure to a small number of stressors. Some inconsistencies with expected pattern that can be explained by various factors.* | *Limited or no studies reporting dependent change in both events following exposure to a specific stressor; and/or significant inconsistencies in empirical support across taxa and species that don’t align with hypothesized AOP* |
| MIE =>KE1: Oxidative Stress leads to Increased oxidized LDL uptake by macrophages | **High** | - Cigarette smoking is associated with increased lipid peroxidation and causes an impairment in antioxidant systems (Solak et al., 2005). - Results demonstrate that relatively stable oxidants in Cigarette smoke extract can pass through the pulmonary alveolar wall into the blood and induce systemic oxidative stress, which most likely facilitates oxidative modification of LDL and endothelial dysfunction, explaining early key events in the development of atherosclerosis (Yamaguchi et al., 2007). - Cigarette smoking (CS) and hypercholesterolemia synergistically impair endothelial function and that their combined presence is associated with increased plasma levels of autoantibodies against oxidized LDL. Long-term smoking potentiates endothelial dysfunction in hypercholesterolemic patients by enhancing the oxidation of LDL (Heitzer, Ylä-Herttuala, et al., 1996; Kunitomo et al., 2009). - Exposure of nonsmoking subjects to secondhand smoke breaks down the serum antioxidant defense, leading to accelerated lipid peroxidation, LDL modification, and accumulation of LDL cholesterol in human macrophages (Valkonen & Kuusi, 1998). CS exposure resulted in significant and dose-dependent increases in lipid peroxides medium levels. Incubation of macrophages with CS-exposed medium resulted in dose-dependent increases in macrophage damage/injury (up to 6-fold), intracellular ROS levels (up to 31%), PON2 activity (up to 2-fold), and macrophage cholesterol content (up to 24%) (Rom & Aviram, 2016; Scheffler et al., 1992). - Lipid peroxidation induced by oxidants present in the gas phase of CS leads to potentially atherogenic changes in lipoproteins (Frei et al., 1991). - Cigarette smoke extract and cigarette smoke components in the same plasma concentration range found in smokers increased the CD36(+)/CD14(+) cell population in human peripheral blood mononuclear cells, increased CD36 expression of human THP1 macrophages, and increased macrophage production of reactive oxygen species (Mehta, Srivastava, et al., 2020; Momeni-Moghaddam et al., 2020; Zhou et al., 2013). - CD36 mRNA and protein expression levels were significantly increased in the cells treated with cigarette smoke extract compared to the control (p<0.001 in mRNA expression levels and p=0.016 in protein expression levels, respectively) (Momeni-Moghaddam et al., 2020). - Cigarette smoke components-induced CD36 expression was suppressed by antioxidants and by specific PKC delta and PPARgamma inhibitors, implicating mechanistic roles for these intermediates (Zhou et al., 2013). - CD36 is a major macrophage receptor for proatherogenic oxidized LDLs mediating lipid uptake, accumulation, and foam cell formation (Silverstein & Febbraio, 2009). - CD36 has the major role in atherosclerotic lesion development in vivo (demonstrated on CD36-apo E double-null mice model (Zhou et al., 2013). - In human macrophages cigarette smoke components increased oxLDL-induced lipid accumulation and foam cell formation, which was prevented by CD36 siRNA (Zhou et al., 2013). Treatment of apoE-/- mice with cigarette smoke components markedly exacerbated inflammatory monocyte levels and atherosclerotic plaque accumulation, effects that were not seen in CD36-/- apoE-/- mice (Zhou et al., 2013). - In mouse bone marrow-derived macrophages (BMDMs) cigarette smoke components promotes oxLDLs phagocytosis that was suppressed by Thioredoxin-interacting protein (TXNIP) si-RNA and ROS inhibitor (Mao et al., 2021). | | |
| KE1=>KE2: Increased oxidized LDL uptake by macrophages leads to Foam cell formation | **High** | - Macrophages expressing scavenger receptors take up oxidized-LDL and differentiate into lipid-laden foam cells. In human THP-1 cells cigarette smoke concentrate exposure increases intracellular ROS levels at all stages of monocyte-to-macrophage-to-foam cell differentiation *in vitro* contributing to initiation and progression of atherosclerosis (Zhou et al., 2013), (Mehta & Dhawan, 2020). - To become lipid-laden foam cells, differentiated THP-1 macrophages were stimulated by ox-LDL treatment at a concentration of 150 μg/ml for 24 h (Mehta & Dhawan, 2020). - In human macrophages, cigarette smoke components increased oxLDL-induced lipid accumulation and foam cell formation, which was prevented by CD36 siRNA (Zhou et al., 2013). | | |
| KE2=>AO: Foam cell formation leads to Plaque progression |  |  |  |  |
| MIE =>KE3: Oxidative Stress leads to Increased pro-inflammatory mediators | **High** | - Cigarette smoke induces NADPH oxidase/ROS-dependent proinflammatory alterations in the vascular endothelium associated with atherosclerosis (Orosz et al., 2007). - In human subjects with coronary atherosclerosis cigarette smoking significantly augmented oxidative stress leading to NLRP3 inflammasome activation and increased production of pro-cytokines IL-1 beta and IL-18 (Mehta, Vijayvergiya, et al., 2020). - Cigarette smoke components stimulate generation of ROS and activation of NLRP3 inflammasome in endothelial vascular smooth muscle cells, human aortic endothelial cells (HAECs) leading to IL-18 and IL-1 secretion (Wu et al., 2018; Yao et al., 2019). - Treating bone marrow-derived macrophages (BMDMs) with cigarette smoke components in vitro led to enhanced lipid phagocytosis, chemotaxis, and increased production of reactive oxygen species (ROS), which activated TXNIP/NLRP3 inflammasome signaling and promoted pyroptosis, as evidenced by caspase-1 cleavage and increased production of IL-1 beta, IL-18, and gasdermin D (Mao et al., 2021). - ROS produced in endothelial cells, leads to the activation of NF-kB, which contributes to the expression of proinflammatory factors, such as iNOS and cytokines TNF-alpha, IL-6, and IL-1 beta (Orosz et al., 2007). - Inhibition of NAD(P)H oxidase (by diphenyleneiodonium and apocynin) or scavenging of H(2)O(2) (by catalase) leads to inhibition of oxidative stress and suppressed the expression of inflammatory mediators (Orosz et al., 2007). | | |
| KE3=>KE4: Increased pro-inflammatory mediators lead to Leukocyte recruitment/ activation | **High** | - Several studies experimentally demonstrated that cigarette smoke-induced oxidative stress promoted proinflammatory factors secretion and monocyte/ macrophage recruitment to the vascular wall leading to vascular inflammation, endothelial damage and dysfunction (Edirisinghe et al., 2008; Mao et al., 2021; Orosz et al., 2007). - Smoking and in vitro cigarette smoke extract (CSE) exposure upregulate vascular expression of inflammatory markers, such as IL-1beta, IL-6, and TNF-alpha and iNOS, which were prevented by apocynin and catalase (inhibitors of oxidative stress). In vitro exposure of cultured carotid arteries and aortas to CSE also increased monocyte adhesiveness to the endothelium (increased monocyte adhesiveness induced by H(2)O(2) and TNF-alpha was shown as positive controls) (Orosz et al., 2007). - In mouse lung, cigarette smoke induced oxidative stress, influx of macrophages and increased secretion of proinflammatory mediators (KC and MCP-1), inhibition of VEGFR-2 augmented CS-induced oxidative stress and inflammatory responses leading to endothelial dysfunction (Edirisinghe et al., 2008). | | |
| KE4=>KE6: Leukocyte recruitment/ activation leads to Endothelial cell dysfunction |  |  |  |  |
| MIE=>KE5: Oxidative Stress leads to NO depletion | **High** | - Multiple studies demonstrated that oxidative stress induced by variuos toxicants (including cigarette smoke) causes NO depletion (Abdelghany et al., 2018; Barua et al., 2003; El-Mahdy et al., 2020). - Cigarette smoking exposure leads to reduced bioavailability of Tetrahydrobiopterin (a required cofactor for NO synthesis by eNOS and has been demonstrated to cause an uncoupling of eNOS thereby generating superoxide instead of NO (Abdelghany et al., 2018; El-Mahdy et al., 2020; Heitzer et al., 2000). - Oxidative stress plays a central role in smoking-mediated dysfunction of NO biosynthesis in endothelial cells. Exposure of bovine pulmonary artery endothelial cells (BPAECs), human pulmonary artery endothelial cells, and rat pulmonary arteries to CS extracts (CSEs) resulted in a large increase in superoxide production leading to decrease in NO bioactivity (Jaimes et al., 2004). - Human coronary artery endothelial cells (HCAECs) incubated with smokers' serum alone showed significantly lower NO production (P<0.05) and eNOS activity (P<0.005) but higher eNOS expression (P<0.005) compared with nonsmokers. In smokers, addition of PEG-SOD, PEG-SOD+PEG-catalase, or tetrahydrobiopterin significantly (P<0.05) improved NO levels and eNOS activity (Barua et al., 2003). - Exposure of aortic endothelial cells (AECs) to cigarette smoke extract resulted in a marked decrease in NO production with concomitant increase in superoxide (O2.-) generation and accumulation of 4-hydroxy-2-nonenal protein adducts (Abdelghany et al., 2018). | | |
| KE5=>KE6: NO depletion leads to Endothelial cell dysfunction | **High** | - Many studies demonstrated that reduced NO bioavailability directly contributes to development of vascular endothelial dysfunction and atherosclerosis (Alp et al., 2004; El-Mahdy et al., 2020). - NO depletion contributes at least in part to endothelial cell dysfunction in chronic smokers (Heitzer et al., 2000). - Cigarette smoke extract increases endothelial superoxide production in bovine pulmonary artery endothelial cells (BPAECs), human pulmonary artery endothelial cells, and rat pulmonary arteries, thereby reducing NO bioactivity and resulting in endothelial dysfunction (Jaimes et al., 2004). - Antioxidant vitamin C markedly improves endothelium-dependent responses in chronic smokers. This observation supports the concept that endothelial dysfunction in chronic smokers is at least in part mediated by enhanced formation of oxygen-derived free radicals (Heitzer, Just, et al., 1996; Puranik & Celermajer, 2003). - Reversal of eNOS uncoupling and NO depletion is achieved by supplementation of tetrahydrobiopterin, which restores NO production and improves cigarette smoke-induced endothelial dysfunction (Alp et al., 2004; Heitzer et al., 2000; Li et al., 2018). | | |
| KE6=>AO: Endothelial cell dysfunction leads to Plaque progression | **High** | - Several studies demonstrate that smoking-induced oxidative stress is associated with endothelial dysfunction, an early stage of atherosclerosis (Heitzer, Just, et al., 1996; Li et al., 2018; Papamichael et al., 2004; Shimosato et al., 2012). - Cigarette smoke components induced endothelial dysfunction and promoted atherosclerotic plaque progression in ApoE-/- mice in vivo (Li et al., 2018), which was partially mediated by the pyroptosis and damage of endothelial cells (Mao et al., 2021; Wu et al., 2018). - In transgenic mouse model with endothelial-targeted overexpression of the rate-limiting enzyme in Tetrahydrobiopterin synthesis, GTP-cyclohydrolase I (GTPCH), was demonstrated that higher aortic Tetrahydrobiopterin levels were associated with reduced endothelial superoxide production and eNOS uncoupling, increased cGMP levels, and preserved NO-mediated endothelium dependent vasorelaxations. Furthermore, aortic root atherosclerotic plaque was significantly reduced in ApoE-KO/GCH-Tg mice compared with ApoE-KO controls (Alp et al., 2004). | | |
| KE5=>KE7: NO depletion leads to Increase, platelet aggregation | **High** | - Several studies underlined the role of NO bioavailability in platelet aggregation (Dangel et al., 2010; Ichiki et al., 1996; Radziwon-Balicka et al., 2017; Takajo et al., 2001). - Chronic smoking impairs platelet-derived nitric oxide (PDNO) bioactivity and augments platelet aggregability (Ichiki et al., 1996; Takajo et al., 2001). - The collagen-induced intraplatelet cGMP was significantly lower in smokers than in nonsmokers. Furthermore, the increase in intraplatelet cGMP level induced by l-arginine was significantly smaller in smokers than in nonsmokers. Because the effect of NO is mediated by cGMP, these findings further support that the NO release during platelet aggregation may be impaired in long-term smokers (Ichiki et al., 1996). - Vitamin C (antioxidant) administration decreased intraplatelet nitrotyrosine production and inhibited platelet aggregation in smokers (Takajo et al., 2001). - Smoking cessation quickly reduced agonist-induced platelet aggregations, intraplatelet nitrotyrosine level, and urinary productions of 8-OHdG and 8-iso-PGF(2alpha). Two weeks of smoking cessation can ameliorate the enhanced platelet aggregability and intraplatelet redox imbalance in long-term smokers, possibly by decreasing oxidative stress (Morita et al., 2005). | | |
| KE7=>AO: Increase, platelet aggregation leads to Plaque progression | **Low** | - There are small experimental data about mechanisms of smoking-induced platelet aggregation promote atherosclerotic plaque progression. - Platelet aggregability along with pack-years of smoking was one of the strongest predictors of atherosclerosis progression (assessed by ultrasonographic examination in a population-based sample of 100 Eastern Finnish men aged 42, 48, 54 or 60 years) (Salonen & Salonen, 1990). - In an animal model study was demonstrated that perfusion of activated platelets increased atherosclerotic lesions formation in Apoe–/– mice. Circulating activated platelets bound to leukocytes, preferentially monocytes, to form platelet–monocyte/leukocyte aggregates, promote monocyte recruitment to atherosclerotic arteries and accelerate the formation of atherosclerotic lesions in Apoe–/– mice (Huo et al., 2003). | | |

## Table A.4. Biomarkers associated with MIE, KEs and AO

| ***MIE, KEs, AO*** | ***Biomarkers*** | ***Assay*** | ***References*** |
| --- | --- | --- | --- |
| MIE: Oxidative Stress | Increased intracellular or vascular ROS (O2-, H202) levels | - O2- production was measured by chemiluminescence of lucigenin (Jaimes et al., 2004) - vascular O2•− level: ethidium bromide fluorescence, lucigenin chemiluminescence (Orosz et al., 2007) - H2O2 production by the cell-permeant oxidative fluorescent indicator dye C-H2DCFDA (5 (and 6)-chloromethyl-2′,7′-dichlorodihydrofluorescein diacetate-acetyl ester) and by modified methods of Werner (Orosz et al., 2007) - flow cytometry (Mehta & Dhawan, 2020)\| cellular and mt ROS by flow cytometry (Mehta, Vijayvergiya, et al., 2020) - ROS Detection Assay Kit, flow cytometry (Mao et al., 2021) - 2′,7′‐dichlorodihydrofluorescein diacetate (H2DCF‐DA) fluorescent probe (Yao et al., 2019) - Reactive Oxygen Species Assay Kit (Beyotime) (Wang et al., 2019) - by fluorescent dye, 5-and 6-carboxy-2′,7′-dichlorodihydrofluorescein diacetate (carboxy-H2DCF-DA), fluorescent microscopy (Edirisinghe et al., 2008) - fluorescence staining using dihydroethidium (El-Mahdy et al., 2020) - Reactive oxygen species assay kit (Wu et al., 2018) | (Edirisinghe et al., 2008; El-Mahdy et al., 2020; Jaimes et al., 2004; Mehta & Dhawan, 2020; Mehta, Vijayvergiya, et al., 2020 2021, Nicotine exacerbates atherosclerosis through a macrophage-mediated endothelial injury pathway; Orosz et al., 2007; Wang et al., 2019; Wu et al., 2018; Yao et al., 2019) |
|  | increase in NADPH oxidase subunits p22phox and gp91phox | - Western blot - immunofluorescence measurements | (El-Mahdy et al., 2020) |
|  | increased serum levels of markers of oxidative stress (3-nitrotyrosine, 3-chlorotyrosine, 8-isoprostane, 8-oxo-2́'-deoxyguanosine, 8-hydroxy-2'-deoxyguanine, F2-isoprostanes) | - commercially available Enzyme-linked Immunosorbent Assay (ELISA) kits (Sincere Biotech, USA) as per manufacturer’s protocol (Mehta, Vijayvergiya, et al., 2020) - stable-isotope-dilution mass-spectrometric assay (Morrow et al., 1995) - HPLC combined with the use of a coulometric electrochemical array detector (HPLC-ECD) (Yamaguchi et al., 2007) - HPLC with electrochemical detection (Kunitomo et al., 2009) - immunochemical detection assay (Takajo et al., 2001) - FACScan (Morita et al., 2005) | (Kunitomo et al., 2009; Mehta, Vijayvergiya, et al., 2020; Morita et al., 2005; Morrow et al., 1995; Takajo et al., 2001; Yamaguchi et al., 2007) |
|  | increased cytotoxicity markers (Lactate dehydrogenase (LDH) | - release of LDH into the culture medium using a commercially available kit (Rom & Aviram, 2016) - LDH assay kit (Wu et al., 2018) | (Rom & Aviram, 2016; Wu et al., 2018) |
|  | increased paraoxonase2 (PON2) activity | lactonase activity was measured using DHC as a substrate | (Rom & Aviram, 2016) |
|  | Decrease in plasma total antioxidant defense (combined capacity of all plasma antioxidants to resist artificially induced peroxidation) | Spectrophotometric assay for total peroxyl radical-trapping potential | (Valkonen & Kuusi, 1998; Valkonen & Kuusi, 2000) |
|  | Decrease in intraplatelet GSH levels | high-performance liquid chromatography with an electrochemical detection system | (Morita et al., 2005; Takajo et al., 2001) |
|  | Increased urinary levels of 8-hydroxy-2'-deoxyguanosine (8-OHdG) and urinary 8-iso-prostaglandin F(2alpha) (8-iso-PGF(2alpha) | - ELISA kit (for 8-OHdG), - gas chromatography/mass spectrometry (for 8-iso-PGF(2alpha) | (Morita et al., 2005) |
|  | Decrease in vitamin E | high performance liquid chromatography | (Scheffler et al., 1992) |
| KE1: Increased oxidized LDL uptake by macrophages | Increased ox-LDL phagocytosis | - flow cytometry (Mao et al., 2021) - Cellular lipoprotein uptake was assessed by measuring lipoprotein cellular degradation. The cell-mediated hydrolysis of oxLDL protein was assessed by determination of trichloroacetic acid-soluble and chloroform-insoluble radioactivity in the incubation medium | (Mao et al., 2021; Zhou et al., 2013) |
|  | increased plasma levels of autoantibodies against oxidized LDL | autoantibody titer assay | (Heitzer, Ylä-Herttuala, et al., 1996) |
|  | Increased total cholesterol | fluoro-enzymatic method | (Kunitomo et al., 2009) |
|  | Increased lipid peroxides and thiobarbituric acid reactive substances (TBARS) | - fluorometry (Kunitomo et al., 2009) - lipid peroxides assay and by the TBARS assay (Rom & Aviram, 2016) - Gas-liquid chromatography, thiobarbituric acid reaction (Valkonen & Kuusi, 2000) - thin-layer chromatography, thiobarbituric Acid Reaction assay (Scheffler et al., 1992; Valkonen & Kuusi, 1998) - HPLC/isoluminol chemiluminescence assay (Frei et al., 1991) | (Frei et al., 1991; Kunitomo et al., 2009; Rom & Aviram, 2016; Scheffler et al., 1992; Valkonen & Kuusi, 1998; Valkonen & Kuusi, 2000) |
| KE2: Foam cell formation | Increased expression levels of surface markers CD14, CD36, CD54 | flow cytometry | (Mehta & Dhawan, 2020; Zhou et al., 2013) |
|  | Increased PKC delta phosphorylation, increased PPAR gamma expression | Immunoblot assays | (Zhou et al., 2013) |
| KE3: Increased pro-inflammatory mediators | NLRP3 inflammasome assembly markers: NLRP3, GSDMD, pro-caspase-1, caspase-1, pro-IL-18, pro-IL-1beta. Increased IL-1beta and IL-18 | - RT-PCR, immunoblotting (Mehta, Vijayvergiya, et al., 2020) - by ELISA assay kits according to the manufacturer's instructions (Mao et al., 2021; Mehta & Dhawan, 2020; Mehta, Srivastava, et al., 2020; Wu et al., 2018; Yao et al., 2019) - Western blot (Yao et al., 2019) | (Mao et al., 2021; Mehta & Dhawan, 2020; Mehta, Srivastava, et al., 2020; Mehta, Vijayvergiya, et al., 2020; Wu et al., 2018; Yao et al., 2019) |
|  | Increased TXNIP expression | real-time PCR | (Mao et al., 2021) |
|  | Increased C-reactive protein (CRP) expression | Western blot | (Yao et al., 2019) |
|  | Increased vascular expression of iNOS, TNF-alpha, IL-1 beta, and IL-6, monocyte chemoattractant protein-1, CXCL9. | - Quantitative RT-PCR (Orosz et al., 2007) - Immunoblot analyses (Zhou et al., 2013) | (Orosz et al., 2007; Zhou et al., 2013) |
| KE4: Leukocyte recruitment/ activation | Increased expression levels of the inflammatory surface markers CD11b, CD14, and CD36 | flow cytometry | (Mehta & Dhawan, 2020; Mehta, Srivastava, et al., 2020) |
|  | Enhanced transcriptional activity of NF-κB | - reporter gene assay by Dual Luciferase Reporter Assay Kit (Promega) - luminometer | (Orosz et al., 2007) |
|  | enhanced monocyte adhesion to the endothelium | microplate-based assay | (Orosz et al., 2007) |
|  | increased macrophage chemotaxis | transwell chamber migration assay | (Mao et al., 2021) |
|  | vascular monocyte infiltration | hematoxylin eosin (H&E) staining, tissue histopathology | (El-Mahdy et al., 2020) |
| KE5: NO depletion | Decreased eNOS expression, decreased eNOS activity | - SDS-PAGE, Immunoblotting (Edirisinghe et al., 2008) - Western blot, Immunohistology (El-Mahdy et al., 2020) - protein activity assay kit (Barua et al., 2003) - eNOS inhibitior treatment (Abdelghany et al., 2018) | (Abdelghany et al., 2018; Barua et al., 2003; Edirisinghe et al., 2008; El-Mahdy et al., 2020) |
|  | decreased NO production | - chemiluminescence method using a NO analyzer (Barua et al., 2003) - Spin-trapping measurement of NO using EPR spectrometer set (Abdelghany et al., 2018) - using commercial kit (Li et al., 2018) | (Abdelghany et al., 2018; Barua et al., 2003; Li et al., 2018) |
|  | impaired platelet-derived nitric oxide (PDNO) | Measurement of the electrical current with an NO-specific electrode (NO meter) | (Ichiki et al., 1996; Morita et al., 2005; Takajo et al., 2001) |
|  | decreased intraplatelet cGMP | radioimmunoassay kit | (Ichiki et al., 1996; Takajo et al., 2001) |
|  | Decreased tetrahydrobiopterin (BH4) | - High-performance liquid chromatography analysis (Abdelghany et al., 2018; El-Mahdy et al., 2020) - ELISA kit (Li et al., 2018) | (Abdelghany et al., 2018; El-Mahdy et al., 2020; Li et al., 2018) |
|  | Decreased expression of GTPCH1 | - Quantitative reverse transcription-polymerase chain reaction (Abdelghany et al., 2018; Li et al., 2018) - Western blot (Li et al., 2018) | (Abdelghany et al., 2018; Li et al., 2018) |
| KE6: Endothelial cell dysfunction | Attenuated forearm blood flow (FBF) and endothelium-dependent relaxation in response to the endothelium-dependent vasodilators | - wire Myograph System-61M (El-Mahdy et al., 2020) - vessel myograph system (Danish Myotechnologies, Aarhus, Denmark) (Li et al., 2018) - venous occlusion plethysmography (Heitzer et al., 2000; Heitzer, Just, et al., 1996; Heitzer, Ylä-Herttuala, et al., 1996) - Flow-mediated dilatation was assessed by B-mode high-resolution ultrasound imaging (Papamichael et al., 2004) - Isometric tension studies of contractile response (Shimosato et al., 2012) | (El-Mahdy et al., 2020; Heitzer et al., 2000; Heitzer, Just, et al., 1996; Heitzer, Ylä-Herttuala, et al., 1996; Li et al., 2018; Papamichael et al., 2004; Shimosato et al., 2012) |
|  | Decreased VEGFR-2 expression and VEGF-induced VEGFR-2 phosphorylation | immunoblotting | (Edirisinghe et al., 2008) |
|  | decreased Akt expression and phosphorylation | Western blot | (El-Mahdy et al., 2020) |
| KE7: Increase, platelet aggregation | Increased platelet aggregability | - collagen-induced platelet aggregation by a platelet aggregometer (Ichiki et al., 1996; Morita et al., 2005; Takajo et al., 2001) - Platelet aggregability in plasma samples was measured as a function of time by the Whole Blood Aggro-Meter model 500 (Salonen & Salonen, 1990) | (Ichiki et al., 1996; Morita et al., 2005; Salonen & Salonen, 1990; Takajo et al., 2001) |
| AO: Plaque progression | Increase of intimal-medial thickness of the carotid artery | - ultrasonographic scanning of carotid arteries (Salonen & Salonen, 1990) - B-mode real-time ultrasound (Howard et al., 1998) | (Howard et al., 1998; Salonen & Salonen, 1990) |
|  | Increase in plaque area | - staining the aortic plaque with Oil Red O, - atherosclerotic lesion sizes were assessed using commercial software (Image Pro Plus 6.0, Cybernetics, USA) | (Li et al., 2018; Mao et al., 2021; Wang et al., 2019) |

## References:

In work

Abdelghany, T. M., Ismail, R. S., Mansoor, F. A., Zweier, J. R., Lowe, F., & Zweier, J. L. (2018). Cigarette smoke constituents cause endothelial nitric oxide synthase dysfunction and uncoupling due to depletion of tetrahydrobiopterin with degradation of GTP cyclohydrolase. *Nitric Oxide*, *76*, 113-121. <https://doi.org/10.1016/j.niox.2018.02.009>

Alp, N. J., McAteer, M. A., Khoo, J., Choudhury, R. P., & Channon, K. M. (2004). Increased endothelial tetrahydrobiopterin synthesis by targeted transgenic GTP-cyclohydrolase I overexpression reduces endothelial dysfunction and atherosclerosis in ApoE-knockout mice. *Arterioscler Thromb Vasc Biol*, *24*(3), 445-450. <https://doi.org/10.1161/01.ATV.0000115637.48689.77>

Ambrose, J. A., & Barua, R. S. (2004). The pathophysiology of cigarette smoking and cardiovascular disease: an update. *J Am Coll Cardiol*, *43*(10), 1731-1737. <https://doi.org/10.1016/j.jacc.2003.12.047>

Arai, H. (2014). Oxidative modification of lipoproteins. *Subcell Biochem*, *77*, 103-114. <https://doi.org/10.1007/978-94-007-7920-4_9>

Barua, R. S., Ambrose, J. A., Srivastava, S., DeVoe, M. C., & Eales-Reynolds, L. J. (2003). Reactive oxygen species are involved in smoking-induced dysfunction of nitric oxide biosynthesis and upregulation of endothelial nitric oxide synthase: an in vitro demonstration in human coronary artery endothelial cells. *Circulation*, *107*(18), 2342-2347. <https://doi.org/10.1161/01.CIR.0000066691.52789.BE>

Bergmann, S., Siekmeier, R., Mix, C., & Jaross, W. (1998). Even moderate cigarette smoking influences the pattern of circulating monocytes and the concentration of sICAM-1. *Respir Physiol*, *114*(3), 269-275. <https://doi.org/10.1016/s0034-5687(98)00098-x>

Checa, J., & Aran, J. M. (2020). Reactive Oxygen Species: Drivers of Physiological and Pathological Processes. *J Inflamm Res*, *13*, 1057-1073. <https://doi.org/10.2147/JIR.S275595>

Chen, J. Y., Ye, Z. X., Wang, X. F., Chang, J., Yang, M. W., Zhong, H. H., . . . Yang, S. L. (2018). Nitric oxide bioavailability dysfunction involves in atherosclerosis. *Biomed Pharmacother*, *97*, 423-428. <https://doi.org/10.1016/j.biopha.2017.10.122>

Chistiakov, D. A., Melnichenko, A. A., Myasoedova, V. A., Grechko, A. V., & Orekhov, A. N. (2017). Mechanisms of foam cell formation in atherosclerosis. *J Mol Med (Berl)*, *95*(11), 1153-1165. <https://doi.org/10.1007/s00109-017-1575-8>

Churg, A., & Cherukupalli, K. (1993). Cigarette smoke causes rapid lipid peroxidation of rat tracheal epithelium. *Int J Exp Pathol*, *74*(2), 127-132.

Crabtree, M. J., & Channon, K. M. (2011). Synthesis and recycling of tetrahydrobiopterin in endothelial function and vascular disease. *Nitric Oxide*, *25*(2), 81-88. <https://doi.org/10.1016/j.niox.2011.04.004>

Csordas, A., & Bernhard, D. (2013). The biology behind the atherothrombotic effects of cigarette smoke. *Nat Rev Cardiol*, *10*(4), 219-230. <https://doi.org/10.1038/nrcardio.2013.8>

Dangel, O., Mergia, E., Karlisch, K., Groneberg, D., Koesling, D., & Friebe, A. (2010). Nitric oxide-sensitive guanylyl cyclase is the only nitric oxide receptor mediating platelet inhibition. *J Thromb Haemost*, *8*(6), 1343-1352. <https://doi.org/10.1111/j.1538-7836.2010.03806.x>

Edirisinghe, I., Yang, S. R., Yao, H., Rajendrasozhan, S., Caito, S., Adenuga, D., . . . Rahman, I. (2008). VEGFR-2 inhibition augments cigarette smoke-induced oxidative stress and inflammatory responses leading to endothelial dysfunction. *FASEB J*, *22*(7), 2297-2310. <https://doi.org/10.1096/fj.07-099481>

El-Mahdy, M. A., Abdelghany, T. M., Hemann, C., Ewees, M. G., Mahgoup, E. M., Eid, M. S., . . . Zweier, J. L. (2020). Chronic cigarette smoke exposure triggers a vicious cycle of leukocyte and endothelial-mediated oxidant stress that results in vascular dysfunction. *Am J Physiol Heart Circ Physiol*, *319*(1), H51-H65. <https://doi.org/10.1152/ajpheart.00657.2019>

Feingold, K. R., Anawalt, B., Boyce, A., Chrousos, G., de Herder, W. W., Dhatariya, K., . . . Wilson, D. P. (2000). Endotext. In. <https://doi.org/NBK343489>

Forgione, M. A., & Loscalzo, J. (2000). Oxidant stress as a critical determinant of endothelial function. *Drug News Perspect*, *13*(9), 523-529. <https://doi.org/10.1358/dnp.2000.13.9.858480>

Frei, B., Forte, T. M., Ames, B. N., & Cross, C. E. (1991). Gas phase oxidants of cigarette smoke induce lipid peroxidation and changes in lipoprotein properties in human blood plasma. Protective effects of ascorbic acid. *Biochem J*, *277 ( Pt 1)*, 133-138. <https://doi.org/10.1042/bj2770133>

Förstermann, U., Xia, N., & Li, H. (2017). Roles of Vascular Oxidative Stress and Nitric Oxide in the Pathogenesis of Atherosclerosis. *Circ Res*, *120*(4), 713-735. <https://doi.org/10.1161/CIRCRESAHA.116.309326>

Gawaz, M., Stellos, K., & Langer, H. F. (2008). Platelets modulate atherogenesis and progression of atherosclerotic plaques via interaction with progenitor and dendritic cells. *J Thromb Haemost*, *6*(2), 235-242. <https://doi.org/10.1111/j.1538-7836.2007.02867.x>

Gimbrone, M. A., & García-Cardeña, G. (2016). Endothelial Cell Dysfunction and the Pathobiology of Atherosclerosis. *Circ Res*, *118*(4), 620-636. <https://doi.org/10.1161/CIRCRESAHA.115.306301>

Grassi, D., Desideri, G., Ferri, L., Aggio, A., Tiberti, S., & Ferri, C. (2010). Oxidative stress and endothelial dysfunction: say NO to cigarette smoking! *Curr Pharm Des*, *16*(23), 2539-2550. <https://doi.org/10.2174/138161210792062867>

Grebe, A., Hoss, F., & Latz, E. (2018). NLRP3 Inflammasome and the IL-1 Pathway in Atherosclerosis. *Circ Res*, *122*(12), 1722-1740. <https://doi.org/10.1161/CIRCRESAHA.118.311362>

Heinecke, J. W., Baker, L., Rosen, H., & Chait, A. (1986). Superoxide-mediated modification of low density lipoprotein by arterial smooth muscle cells. *J Clin Invest*, *77*(3), 757-761. <https://doi.org/10.1172/JCI112371>

Heitzer, T., Brockhoff, C., Mayer, B., Warnholtz, A., Mollnau, H., Henne, S., . . . Münzel, T. (2000). Tetrahydrobiopterin improves endothelium-dependent vasodilation in chronic smokers : evidence for a dysfunctional nitric oxide synthase. *Circ Res*, *86*(2), E36-41. <https://doi.org/10.1161/01.res.86.2.e36>

Heitzer, T., Just, H., & Münzel, T. (1996). Antioxidant vitamin C improves endothelial dysfunction in chronic smokers. *Circulation*, *94*(1), 6-9. <https://doi.org/10.1161/01.cir.94.1.6>

Heitzer, T., Ylä-Herttuala, S., Luoma, J., Kurz, S., Münzel, T., Just, H., . . . Drexler, H. (1996). Cigarette smoking potentiates endothelial dysfunction of forearm resistance vessels in patients with hypercholesterolemia. Role of oxidized LDL. *Circulation*, *93*(7), 1346-1353. <https://doi.org/10.1161/01.cir.93.7.1346>

Howard, G., Wagenknecht, L. E., Burke, G. L., Diez-Roux, A., Evans, G. W., McGovern, P., . . . Tell, G. S. (1998). Cigarette smoking and progression of atherosclerosis: The Atherosclerosis Risk in Communities (ARIC) Study. *JAMA*, *279*(2), 119-124. <https://doi.org/10.1001/jama.279.2.119>

Huo, Y., & Ley, K. F. (2004). Role of platelets in the development of atherosclerosis. *Trends Cardiovasc Med*, *14*(1), 18-22. <https://doi.org/10.1016/j.tcm.2003.09.007>

Huo, Y., Schober, A., Forlow, S. B., Smith, D. F., Hyman, M. C., Jung, S., . . . Ley, K. (2003). Circulating activated platelets exacerbate atherosclerosis in mice deficient in apolipoprotein E. *Nat Med*, *9*(1), 61-67. <https://doi.org/10.1038/nm810>

Ichiki, K., Ikeda, H., Haramaki, N., Ueno, T., & Imaizumi, T. (1996). Long-term smoking impairs platelet-derived nitric oxide release. *Circulation*, *94*(12), 3109-3114. <https://doi.org/10.1161/01.cir.94.12.3109>

Jaimes, E. A., DeMaster, E. G., Tian, R. X., & Raij, L. (2004). Stable compounds of cigarette smoke induce endothelial superoxide anion production via NADPH oxidase activation. *Arterioscler Thromb Vasc Biol*, *24*(6), 1031-1036. <https://doi.org/10.1161/01.ATV.0000127083.88549.58>

Kattoor, A. J., Pothineni, N. V. K., Palagiri, D., & Mehta, J. L. (2017). Oxidative Stress in Atherosclerosis. *Curr Atheroscler Rep*, *19*(11), 42. <https://doi.org/10.1007/s11883-017-0678-6>

Kunitomo, M., Yamaguchi, Y., Kagota, S., Yoshikawa, N., Nakamura, K., & Shinozuka, K. (2009). Biochemical evidence of atherosclerosis progression mediated by increased oxidative stress in apolipoprotein E-deficient spontaneously hyperlipidemic mice exposed to chronic cigarette smoke. *J Pharmacol Sci*, *110*(3), 354-361. <https://doi.org/10.1254/jphs.09100fp>

Li, J., Liu, S., Cao, G., Sun, Y., Chen, W., Dong, F., . . . Zhang, W. (2018). Nicotine induces endothelial dysfunction and promotes atherosclerosis via GTPCH1. *J Cell Mol Med*, *22*(11), 5406-5417. <https://doi.org/10.1111/jcmm.13812>

Makhoul, S., Walter, E., Pagel, O., Walter, U., Sickmann, A., Gambaryan, S., . . . Jurk, K. (2018). Effects of the NO/soluble guanylate cyclase/cGMP system on the functions of human platelets. *Nitric Oxide*, *76*, 71-80. <https://doi.org/10.1016/j.niox.2018.03.008>

Malekmohammad, K., Sewell, R. D. E., & Rafieian-Kopaei, M. (2019). Antioxidants and Atherosclerosis: Mechanistic Aspects. *Biomolecules*, *9*(8). <https://doi.org/10.3390/biom9080301>

Mao, C., Li, D., Zhou, E., Zhang, J., Wang, C., & Xue, C. (2021). Nicotine exacerbates atherosclerosis through a macrophage-mediated endothelial injury pathway. *Aging (Albany NY)*, *13*(5), 7627-7643. <https://doi.org/10.18632/aging.202660>

Martin-Ventura, J. L., Rodrigues-Diez, R., Martinez-Lopez, D., Salaices, M., Blanco-Colio, L. M., & Briones, A. M. (2017). Oxidative Stress in Human Atherothrombosis: Sources, Markers and Therapeutic Targets. *Int J Mol Sci*, *18*(11). <https://doi.org/10.3390/ijms18112315>

Mehta, S., & Dhawan, V. (2020). Exposure of cigarette smoke condensate activates NLRP3 inflammasome in THP-1 cells in a stage-specific manner: An underlying role of innate immunity in atherosclerosis. *Cell Signal*, *72*, 109645. <https://doi.org/10.1016/j.cellsig.2020.109645>

Mehta, S., Srivastava, N., Bhatia, A., & Dhawan, V. (2020). Exposure of cigarette smoke condensate activates NLRP3 inflammasome in vitro and in vivo: A connotation of innate immunity and atherosclerosis. *Int Immunopharmacol*, *84*, 106561. <https://doi.org/10.1016/j.intimp.2020.106561>

Mehta, S., Vijayvergiya, R., & Dhawan, V. (2020). Activation of NLRP3 inflammasome assembly is associated with smoking status of patients with coronary artery disease. *Int Immunopharmacol*, *87*, 106820. <https://doi.org/10.1016/j.intimp.2020.106820>

Messner, B., & Bernhard, D. (2014). Smoking and cardiovascular disease: mechanisms of endothelial dysfunction and early atherogenesis. *Arterioscler Thromb Vasc Biol*, *34*(3), 509-515. <https://doi.org/10.1161/ATVBAHA.113.300156>

Momeni-Moghaddam, M. A., Asadikaram, G., Nematollahi, M. H., Esmaeili Tarzi, M., Faramarz-Gaznagh, S., Mohammadpour-Gharehbagh, A., & Kazemi Arababadi, M. (2020). Effects of Cigarette Smoke and Opium on the Expression of CD9, CD36, and CD68 at mRNA and Protein Levels in Human Macrophage Cell Line THP-1. *Iran J Allergy Asthma Immunol*, *19*(1), 45-55. <https://doi.org/10.18502/ijaai.v19i1.2417>

Morita, H., Ikeda, H., Haramaki, N., Eguchi, H., & Imaizumi, T. (2005). Only two-week smoking cessation improves platelet aggregability and intraplatelet redox imbalance of long-term smokers. *J Am Coll Cardiol*, *45*(4), 589-594. <https://doi.org/10.1016/j.jacc.2004.10.061>

Morrow, J. D., Frei, B., Longmire, A. W., Gaziano, J. M., Lynch, S. M., Shyr, Y., . . . Roberts, L. J. (1995). Increase in circulating products of lipid peroxidation (F2-isoprostanes) in smokers. Smoking as a cause of oxidative damage. *N Engl J Med*, *332*(18), 1198-1203. <https://doi.org/10.1056/NEJM199505043321804>

Mury, P., Chirico, E. N., Mura, M., Millon, A., Canet-Soulas, E., & Pialoux, V. (2018). Oxidative Stress and Inflammation, Key Targets of Atherosclerotic Plaque Progression and Vulnerability: Potential Impact of Physical Activity. *Sports Med*, *48*(12), 2725-2741. <https://doi.org/10.1007/s40279-018-0996-z>

Münzel, T., Hahad, O., Kuntic, M., Keaney, J. F., Deanfield, J. E., & Daiber, A. (2020). Effects of tobacco cigarettes, e-cigarettes, and waterpipe smoking on endothelial function and clinical outcomes. *Eur Heart J*, *41*(41), 4057-4070. <https://doi.org/10.1093/eurheartj/ehaa460>

Orekhov, A. N. (2018). LDL and foam cell formation as the basis of atherogenesis. *Curr Opin Lipidol*, *29*(4), 279-284. <https://doi.org/10.1097/MOL.0000000000000525>

Orosz, Z., Csiszar, A., Labinskyy, N., Smith, K., Kaminski, P. M., Ferdinandy, P., . . . Ungvari, Z. (2007). Cigarette smoke-induced proinflammatory alterations in the endothelial phenotype: role of NAD(P)H oxidase activation. *Am J Physiol Heart Circ Physiol*, *292*(1), H130-139. <https://doi.org/10.1152/ajpheart.00599.2006>

Papamichael, C., Karatzis, E., Karatzi, K., Aznaouridis, K., Papaioannou, T., Protogerou, A., . . . Mavrikakis, M. (2004). Red wine's antioxidants counteract acute endothelial dysfunction caused by cigarette smoking in healthy nonsmokers. *Am Heart J*, *147*(2), E5. <https://doi.org/10.1016/S0002>

Peluso, I., Morabito, G., Urban, L., Ioannone, F., & Serafini, M. (2012). Oxidative stress in atherosclerosis development: the central role of LDL and oxidative burst. *Endocr Metab Immune Disord Drug Targets*, *12*(4), 351-360. <https://doi.org/10.2174/187153012803832602>

Puranik, R., & Celermajer, D. S. (2003). Smoking and endothelial function. *Prog Cardiovasc Dis*, *45*(6), 443-458. <https://doi.org/10.1053/pcad.2003.YPCAD13>

Qian, Z., Zhao, Y., Wan, C., Deng, Y., Zhuang, Y., Xu, Y., . . . Bao, Z. (2021). Pyroptosis in the Initiation and Progression of Atherosclerosis. *Front Pharmacol*, *12*, 652963. <https://doi.org/10.3389/fphar.2021.652963>

Radziwon-Balicka, A., Lesyk, G., Back, V., Fong, T., Loredo-Calderon, E. L., Dong, B., . . . Jurasz, P. (2017). Differential eNOS-signalling by platelet subpopulations regulates adhesion and aggregation. *Cardiovasc Res*, *113*(14), 1719-1731. <https://doi.org/10.1093/cvr/cvx179>

Rom, O., & Aviram, M. (2016). Paraoxsonase2 (PON2) and oxidative stress involvement in pomegranate juice protection against cigarette smoke-induced macrophage cholesterol accumulation. *Chem Biol Interact*, *259*(Pt B), 394-400. <https://doi.org/10.1016/j.cbi.2016.05.009>

Sakakura, K., Nakano, M., Otsuka, F., Ladich, E., Kolodgie, F. D., & Virmani, R. (2013). Pathophysiology of atherosclerosis plaque progression. *Heart Lung Circ*, *22*(6), 399-411. <https://doi.org/10.1016/j.hlc.2013.03.001>

Salonen, R., & Salonen, J. T. (1990). Progression of carotid atherosclerosis and its determinants: a population-based ultrasonography study. *Atherosclerosis*, *81*(1), 33-40. <https://doi.org/10.1016/0021-9150(90)90056-o>

Scheffler, E., Wiest, E., Woehrle, J., Otto, I., Schulz, I., Huber, L., . . . Dresel, H. A. (1992). Smoking influences the atherogenic potential of low-density lipoprotein. *Clin Investig*, *70*(3-4), 263-268. <https://doi.org/10.1007/BF00184660>

Schächinger, V., & Zeiher, A. M. (2000). Atherosclerosis-associated endothelial dysfunction. *Z Kardiol*, *89 Suppl 9*, IX/70-74. <https://doi.org/10.1007/s003920070033>

Shimosato, T., Geddawy, A., Tawa, M., Imamura, T., & Okamura, T. (2012). Chronic administration of nicotine-free cigarette smoke extract impaired endothelium-dependent vascular relaxation in rats via increased vascular oxidative stress. *J Pharmacol Sci*, *118*(2), 206-214. <https://doi.org/10.1254/jphs.11187fp>

Siasos, G., Tsigkou, V., Kokkou, E., Oikonomou, E., Vavuranakis, M., Vlachopoulos, C., . . . Tousoulis, D. (2014). Smoking and atherosclerosis: mechanisms of disease and new therapeutic approaches. *Curr Med Chem*, *21*(34), 3936-3948. <https://doi.org/10.2174/092986732134141015161539>

Silverstein, R. L., & Febbraio, M. (2009). CD36, a scavenger receptor involved in immunity, metabolism, angiogenesis, and behavior. *Sci Signal*, *2*(72), re3. <https://doi.org/10.1126/scisignal.272re3>

Solak, Z. A., Kabaroğlu, C., Cok, G., Parildar, Z., Bayindir, U., Ozmen, D., & Bayindir, O. (2005). Effect of different levels of cigarette smoking on lipid peroxidation, glutathione enzymes and paraoxonase 1 activity in healthy people. *Clin Exp Med*, *5*(3), 99-105. <https://doi.org/10.1007/s10238-005-0072-5>

Spagnoli, L. G., Bonanno, E., Sangiorgi, G., & Mauriello, A. (2007). Role of inflammation in atherosclerosis. *J Nucl Med*, *48*(11), 1800-1815. <https://doi.org/10.2967/jnumed.107.038661>

Steinberg, D., Parthasarathy, S., Carew, T. E., Khoo, J. C., & Witztum, J. L. (1989). Beyond cholesterol. Modifications of low-density lipoprotein that increase its atherogenicity. *N Engl J Med*, *320*(14), 915-924. <https://doi.org/10.1056/NEJM198904063201407>

Steinhubl, S. R., & Moliterno, D. J. (2005). The role of the platelet in the pathogenesis of atherothrombosis. *Am J Cardiovasc Drugs*, *5*(6), 399-408. <https://doi.org/10.2165/00129784-200505060-00007>

Sukhovershin, R. A., Yepuri, G., & Ghebremariam, Y. T. (2015). Endothelium-Derived Nitric Oxide as an Antiatherogenic Mechanism: Implications for Therapy. *Methodist Debakey Cardiovasc J*, *11*(3), 166-171. <https://doi.org/10.14797/mdcj-11-3-166>

Takajo, Y., Ikeda, H., Haramaki, N., Murohara, T., & Imaizumi, T. (2001). Augmented oxidative stress of platelets in chronic smokers. Mechanisms of impaired platelet-derived nitric oxide bioactivity and augmented platelet aggregability. *J Am Coll Cardiol*, *38*(5), 1320-1327. <https://doi.org/10.1016/s0735-1097(01)01583-2>

Talib, J., Kwan, J., Suryo Rahmanto, A., Witting, P. K., & Davies, M. J. (2014). The smoking-associated oxidant hypothiocyanous acid induces endothelial nitric oxide synthase dysfunction. *Biochem J*, *457*(1), 89-97. <https://doi.org/10.1042/BJ20131135>

Tousoulis, D., Oikonomou, E., Economou, E. K., Crea, F., & Kaski, J. C. (2016). Inflammatory cytokines in atherosclerosis: current therapeutic approaches. *Eur Heart J*, *37*(22), 1723-1732. <https://doi.org/10.1093/eurheartj/ehv759>

Valkonen, M., & Kuusi, T. (1998). Passive smoking induces atherogenic changes in low-density lipoprotein. *Circulation*, *97*(20), 2012-2016. <https://doi.org/10.1161/01.cir.97.20.2012>

Valkonen, M. M., & Kuusi, T. (2000). Vitamin C prevents the acute atherogenic effects of passive smoking. *Free Radic Biol Med*, *28*(3), 428-436. <https://doi.org/10.1016/s0891-5849(99)00260-9>

Viles-Gonzalez, J. F., Fuster, V., & Badimon, J. J. (2004). Atherothrombosis: a widespread disease with unpredictable and life-threatening consequences. *Eur Heart J*, *25*(14), 1197-1207. <https://doi.org/10.1016/j.ehj.2004.03.011>

Wang, L., & Tang, C. (2020). Targeting Platelet in Atherosclerosis Plaque Formation: Current Knowledge and Future Perspectives. *Int J Mol Sci*, *21*(24). <https://doi.org/10.3390/ijms21249760>

Wang, W., Zhao, T., Geng, K., Yuan, G., Chen, Y., & Xu, Y. (2021). Smoking and the Pathophysiology of Peripheral Artery Disease. *Front Cardiovasc Med*, *8*, 704106. <https://doi.org/10.3389/fcvm.2021.704106>

Wang, Z., Liu, B., Zhu, J., Wang, D., & Wang, Y. (2019). Nicotine-mediated autophagy of vascular smooth muscle cell accelerates atherosclerosis via nAChRs/ROS/NF-κB signaling pathway. *Atherosclerosis*, *284*, 1-10. <https://doi.org/10.1016/j.atherosclerosis.2019.02.008>

Witztum, J. L. (1994). The oxidation hypothesis of atherosclerosis. *Lancet*, *344*(8925), 793-795. <https://doi.org/10.1016/s0140-6736(94)92346-9>

Wu, X., Zhang, H., Qi, W., Zhang, Y., Li, J., Li, Z., . . . Yang, B. (2018). Nicotine promotes atherosclerosis via ROS-NLRP3-mediated endothelial cell pyroptosis. *Cell Death Dis*, *9*(2), 171. <https://doi.org/10.1038/s41419-017-0257-3>

Yamaguchi, Y., Haginaka, J., Morimoto, S., Fujioka, Y., & Kunitomo, M. (2005). Facilitated nitration and oxidation of LDL in cigarette smokers. *Eur J Clin Invest*, *35*(3), 186-193. <https://doi.org/10.1111/j.1365-2362.2005.01472.x>

Yamaguchi, Y., Nasu, F., Harada, A., & Kunitomo, M. (2007). Oxidants in the gas phase of cigarette smoke pass through the lung alveolar wall and raise systemic oxidative stress. *J Pharmacol Sci*, *103*(3), 275-282. <https://doi.org/10.1254/jphs.fp0061055>

Yao, Y., Mao, J., Xu, S., Zhao, L., Long, L., Chen, L., . . . Lu, S. (2019). Rosmarinic acid inhibits nicotine-induced C-reactive protein generation by inhibiting NLRP3 inflammasome activation in smooth muscle cells. *J Cell Physiol*, *234*(2), 1758-1767. <https://doi.org/10.1002/jcp.27046>

Yokoyama, M. (2004). Oxidant stress and atherosclerosis. *Curr Opin Pharmacol*, *4*(2), 110-115. <https://doi.org/10.1016/j.coph.2003.12.004>

Yu, X. H., Fu, Y. C., Zhang, D. W., Yin, K., & Tang, C. K. (2013). Foam cells in atherosclerosis. *Clin Chim Acta*, *424*, 245-252. <https://doi.org/10.1016/j.cca.2013.06.006>

Zhou, M. S., Chadipiralla, K., Mendez, A. J., Jaimes, E. A., Silverstein, R. L., Webster, K., & Raij, L. (2013). Nicotine potentiates proatherogenic effects of oxLDL by stimulating and upregulating macrophage CD36 signaling. *Am J Physiol Heart Circ Physiol*, *305*(4), H563-574. <https://doi.org/10.1152/ajpheart.00042.2013>
